# Antimicrobial Resistance in Invasive Bacterial Infections in Hospitalized Children, Cambodia, 2007–2016

**DOI:** 10.3201/eid2405.171830

**Published:** 2018-05

**Authors:** Andrew Fox-Lewis, Junko Takata, Thyl Miliya, Yoel Lubell, Sona Soeng, Poda Sar, Kolthida Rith, Gregor McKellar, Vanaporn Wuthiekanun, Erin McGonagle, Nicole Stoesser, Catrin E. Moore, Christopher M. Parry, Claudia Turner, Nicholas P.J. Day, Ben S. Cooper, Paul Turner

**Affiliations:** University of Oxford, Oxford, UK (A. Fox-Lewis, J. Takata, Y. Lubell, N. Stoesser, C.E. Moore, C. Turner, N.P.J. Day, B.S. Cooper, P. Turner);; Angkor Hospital for Children, Siem Reap, Cambodia (A. Fox-Lewis, T. Miliya, S. Soeng, P. Sar, K. Rith, G. McKellar, C. Turner, P. Turner);; Cambodia-Oxford Medical Research Unit, Siem Reap (A. Fox-Lewis, T. Miliya, S. Soeng, P. Sar, K. Rith, C. Turner, P. Turner);; Mahidol-Oxford Tropical Medicine Research Unit, Bangkok, Thailand (Y. Lubell, V. Wuthiekanun, C.E. Moore, N.P.J. Day, B.S. Cooper);; University of Colorado, Aurora, Colorado, USA (E. McGonagle);; Liverpool School of Tropical Medicine, Liverpool, UK (C.M. Parry);; Nagasaki University, Nagasaki, Japan (C.M. Parry)

**Keywords:** antimicrobial resistance, Cambodia, children, bacteria, invasive infections, hospital

## Abstract

To determine trends, mortality rates, and costs of antimicrobial resistance in invasive bacterial infections in hospitalized children, we analyzed data from Angkor Hospital for Children, Siem Reap, Cambodia, for 2007–2016. A total of 39,050 cultures yielded 1,341 target pathogens. Resistance rates were high; 82% each of *Escherichia coli* and *Klebsiella pneumoniae* isolates were multidrug resistant. Hospital-acquired isolates were more often resistant than community-acquired isolates; resistance trends over time were heterogeneous. *K. pneumoniae* isolates from neonates were more likely than those from nonneonates to be resistant to ampicillin–gentamicin and third-generation cephalosporins. In patients with community-acquired gram-negative bacteremia, third-generation cephalosporin resistance was associated with increased mortality rates, increased intensive care unit admissions, and 2.26-fold increased healthcare costs among survivors. High antimicrobial resistance in this setting is a threat to human life and the economy. In similar low-resource settings, our methods could be reproduced as a robust surveillance model for antimicrobial resistance.

Worldwide, invasive bacterial infections are a leading cause of childhood deaths, mostly in low- and middle-income countries (*1*). Management of such infections is threatened by the rising prevalence of antimicrobial resistance (AMR), particularly among neonates (*2*). However, data on AMR in invasive bacterial infections in children from low- and middle-income countries are scarce (*3*–*6*).

To combat the global threat of AMR, improved surveillance to detect emerging and long-term resistance trends is vital (*7*). Several global initiatives, such as the Fleming Fund, have been recently established to improve laboratory capacity in low- and middle-income countries (*7*,*8*), and the World Health Organization (WHO) Global Antimicrobial Resistance Surveillance System (GLASS) (*9*) has targeted 6 invasive pathogens for routine antimicrobial resistance surveillance: *Escherichia coli*, *Klebsiella pneumoniae*, *Acinetobacter baumannii*, *Salmonella* spp., *Staphylococcus aureus*, and *Streptococcus pneumoniae*. Monitoring resistance in these pathogens is particularly important for invasive bacterial infections in children in low- and middle-income countries, where most treatment is empirically prescribed and must be based on reliable contemporaneous resistance data to be effective.

Recent systematic reviews of AMR in invasive bacterial infections in children highlight the paucity of data available and do not report temporal resistance trends (*5*,*6*). In addition, although recent studies indicate excess deaths caused by AMR in low- and middle-income countries (*10*), there is limited evidence describing the economic and mortality burden of resistance at the patient level, particularly among children.

We analyzed 10 years of continuous AMR surveillance data for invasive bacterial infections in children from a sentinel surveillance site in Cambodia and describe resistance trends over time, by age group, and by site of acquisition (community or hospital). To evaluate the excess deaths and cost burden associated with third-generation cephalosporin resistance in community-acquired gram-negative bacteremia in hospitalized children, we analyzed patient-level data.

## Methods

### Study Design and Sample Selection

Angkor Hospital for Children is an ≈100-bed nongovernmental hospital in Siem Reap, Cambodia. Of children admitted, around two thirds reside in Siem Reap Province (*11*), where the incidence of poverty exceeds 50% (*12*); 93% are admitted from the community and 7% are transferred from another hospital (P. Turner, unpub data). Because this hospital has no maternity/obstetric ward, all children are born outside the hospital. Blood cultures are routinely taken from febrile (axillary temperature >37.5°C) hospitalized patients, according to clinical algorithms, at no patient cost. 

We reviewed hospital microbiology data for 2007–2016 and extracted AMR data for selected blood culture and cerebrospinal fluid (CSF) culture isolates. Target organisms consisted of the 6 GLASS blood culture priority pathogens (*9*), *Neisseria meningitidis* (a vaccine-preventable pathogen), and non-GLASS pathogens for which >30 organisms were isolated over the study period. We included in the study the first isolate of a given organism per patient per 14-day infection episode, except for *Salmonella* spp., for which we included only the first isolate per patient to avoid double counting potential relapses. Clinical data were extracted from hospital patient records. The study was approved by the Angkor Hospital for Children Institutional Review Board (AHC-IRB, 0185-17) and the Oxford Tropical Research Ethics Committee (OxTREC, 508-17).

### Procedures and AMR Reporting

We processed blood and CSF culture specimens as described elsewhere (*11*,*13*). Antimicrobial susceptibility testing was undertaken by disk diffusion and Etest MIC, according to Clinical and Laboratory Standards Institute guidelines (*14*) (Technical Appendix Methods and Table 1). Resistance proportions are reported as number of resistant isolates/number of isolates tested.

### Outcome Analyses

We included in patient outcome analyses community-acquired monomicrobial *Enterobacteriaceae* (excluding *Salmonellae*) and *A. baumannii* bacteremia. These pathogens represent common causes of sepsis in children worldwide where third-generation cephalosporins would be a first-line/empiric treatment. We obtained clinical and costing data from hospital records and calculated cost per patient as admission cost plus antimicrobial costs.

### Statistical Analyses

We treated isolates from specimens taken within 48 hours of admission as community-acquired infections and after 48 hours as hospital-acquired infections. However, *Salmonella enterica* serotypes Typhi and Paratyphi and *Burkholderia pseudomallei* isolates were always considered community-acquired infections. To ensure sufficient data per period, we grouped isolates into 2-year blocks. We assessed associations between resistance and year of isolation, patient age group, and site of acquisition (community vs. hospital) by univariable and multivariable logistic regression. Multivariable models included all variables. According to assessment of model fit by calculation of Akaike information criterion and plotting of observed versus predicted data, we considered time (year of isolation) a factor unless otherwise stated.

For the outcome analyses, we used univariable analysis to compare variables by third-generation cephalosporin resistance status and patient outcome by using the Mann-Whitney-Wilcoxon rank-sum test for continuous variables and the χ^2^ test with Yates correction for categorical variables. For multivariable logistic regression, outcome variables were hospital deaths and intensive care unit (ICU) admissions, and covariates were resistance, age group, age <10 years, malnutrition, sex, and organism type (*Enterobacteriaceae* vs. *A. baumannii*). We conducted multivariable linear regression by using admission duration and cost for survivors as outcome variables and using the same covariates. The linear model variables were log transformed, and results are presented with log- and back-transformed coefficients, which is interpreted as a multiplicative rather than an additive model. Analyses were undertaken by using the R statistical package (*15*).

## Results

During the 10-year study period, 39,050 sterile site samples were collected for culture: 36,358 (93.1%) blood and 2,692 (6.9%) CSF (Technical Appendix Figure 1). The sampling rate, indicated by the blood culture:hospital admission ratio, rose throughout the study period as utility of the clinical microbiology service increased. Approximately 1 blood culture was sent for every 3 admissions in 2007 (1,293 blood cultures:3,829 admissions), rising to 1 blood culture per admission in 2013 (5,294 blood cultures:5,208 admissions) and subsequently remaining stable (Technical Appendix Figure 2). From 2012 through 2016, the proportion of blood cultures from neonates rose from 9.1% to 21.2%, and the proportion from children >5 years of age dropped from 35.4% to 22.4% (Technical Appendix Table 2).

Of the 39,050 specimens collected, 3,666 (9.4%) were culture positive, yielding 4,028 isolates. Skin organism contamination was identified in 1,937 (5.3%) blood cultures. Clearly pathogenic bacteria comprised 37.5% (1,512) of isolates grown, 9.1% (366) were of uncertain significance, and 53.4% (2,150) were designated skin contaminants. A total of 1,341 target organisms met inclusion criteria; 1,088 (81.1%) were GLASS pathogens and 253 (18.9%) were non-GLASS pathogens. GLASS pathogens were *Salmonella* spp. (408, 30.4%); *S. aureus* (186, 13.9%); *S. pneumoniae* (166, 12.4%); *K. pneumoniae* (146, 10.9%); *E. coli* (107, 8.0%); and *A. baumannii* (75, 5.6%).

## Overall AMR Rates 

Overall AMR rates were high, especially among gram-negative GLASS organisms (Table 1). Ampicillin–gentamicin resistance (resistance to both agents) was detected in 62.1% (90/145) of *K. pneumoniae* isolates and 47.2% (50/106) of *E. coli* isolates. Third-generation cephalosporin resistance was detected in 78.8% (115/146) of *K. pneumoniae* isolates, 49.5% (53/107) of *E. coli* isolates, and 93.3% (70/75) of *A. baumannii* isolates; multidrug resistance in these 3 organisms was 81.8% (108/132), 82.1% (69/84), and 93.3% (70/75), respectively. Carbapenem resistance was uncommon: <1% of *K. pneumoniae* (1/142) and *E. coli* isolates (0/98) and 13.5% (10/74) of *A. baumannii* isolates were resistant.

**Table 1:** Resistance proportions by year of isolation for the 1,088 Global Antimicrobial Resistance Surveillance System pathogens isolated from children at Angkor Hospital for Children, Siem Reap, Cambodia, 2007–2016*

Pathogen, resistance type	No. isolates resistant/no. tested (%)	Year of isolation				
		2007–2008	2009–2010	2011–2012	2013–2014	2015–2016
Gram-negative						
* Klebsiella pneumoniae*	146	11	17	56	42	20
AMP–GEN†	90/145 (62.1)	5/11 (45.5)	10/16 (62.5)	46/56 (82.1)	26/42 (61.9)	3/20 (15.0)
3GC	115/146 (78.8)	8/11 (72.7)	13/17 (76.5)	50/56 (89.3)	37/42 (88.1)	7/20 (35.0)
Carbapenem	1/142 (0.7)	0/8	0/16	0/56	1/42 (2.4)	0/20
Multidrug	108/132 (81.8)	8/8 (100)	12/12 (100)	45/50 (90.0)	36/42 (85.7)	7/20 (35.0)
* Escherichia coli*	107	12	22	21	30	22
AMP–GEN	50/106 (47.2)	4/12 (33.3)	12/21 (57.1)	8/21 (38.1)	17/30 (56.7)	9/22 (40.9)
AMP	101/107 (94.4)	10/12 (83.3)	21/22 (95.5)	21/21 (100)	28/30 (93.3)	21/22 (95.5)
GEN	51/106 (48.1)	4/12 (33.3)	12/21 (57.1)	8/21 (38.1)	18/30 (60.0)	9/22 (40.9)
3GC	53/107 (49.5)	3/12 (25.0)	11/22 (50.0)	11/21 (52.4)	16/30 (53.3)	12/22 (54.5)
Carbapenem	0/98	0/3	0/22	0/21	0/30	0/22
Multidrug	69/84 (82.1)	3/3 (100)	13/13 (100)	15/16 (93.8)	23/30 (76.7)	15/22 (68.2)
* Acinetobacter baumannii*	75	2	7	30	27	9
3GC	70/75 (93.3)	2/2 (100)	6/7 (85.7)	27/30 (90.0)	27/27 (100)	8/9 (88.9)
Carbapenem	10/74 (13.5)	1/2 (50.0)	1/6 (16.7)	5/30 (16.7)	3/27 (11.1)	0/9
Multidrug	21/71 (29.6)	1/2 (50.0)	2/6 (33.3)	9/27 (33.3)	8/27 (29.6)	1/9 (11.1)
*Salmonella* Typhi	323	44	51	146	40	42
FQ	308/322 (95.7)	39/44 (88.6)	48/51 (94.1)	139/145 (95.9)	40/40 (100)	42/42 (100)
CRO	1/173 (0.6)	0/44	1/21 (4.8)	0/26	0/40	0/42
MDR	270/314 (86.0)	31/41 (75.6)	39/47 (83.0)	134/144 (93.1)	35/40 (87.5)	31/42 (73.8)
FQ and multidrug	266/313 (85.0)	30/41 (73.2)	38/47 (80.9)	132/143 (92.3)	35/40 (87.5)	31/42 (73.8)
*Salmonella* Paratyphi A	44	3	0	0	35	6
FQ	10/44 (22.7)	3/3 (100)			4/35 (11.4)	3/6 (50.0)
CRO	0/44	0/3			0/35	0/6
MDR	0/43	0/2			0/35	0/6
FQ and multidrug	0/0	0/0			0/0	0/0
Non-Typhoid *Salmonellae*	41	7	4	7	9	14
FQ	26/41 (63.4)	4/7 (57.1)	2/4 (50.0)	4/7 (57.1)	6/9 (66.7)	10/14 (71.4)
CRO	3/37 (8.1)	0/7	0/4	1/3 (33.3)	0/9	2/14 (14.3)
Multidrug	9/39 (23.1)	3/7 (42.9)	1/2 (50.0)	2/7 (28.6)	2/9 (22.2)	1/14 (7.1)
FQ and multidrug	5/39 (12.8)	3/7 (42.9)	1/2 (50.0)	0/7	0/9	1/14 (7.1)
Gram-positive						
* Staphylococcus aureus*	186	26	38	43	42	37
MET	24/185 (13.0)	3/26 (11.5)	4/38 (10.5)	8/42 (19.0)	3/42 (7.1)	6/37 (16.2)
VAN	0/9	0/0	0/0	0/0	0/3	0/6
* Streptococcus pneumoniae*	166	17	36	40	41	32
Penicillin	73/144 (50.7)	5/9 (55.6)	10/23 (43.5)	16/39 (41.0)	20/41 (48.8)	22/32 (68.8)
MAC/LIN	49/165 (29.7)	5/17 (29.4)	10/35 (28.6)	12/40 (30.0)	11/41 (26.8)	11/32 (34.4)
MDR	63/93 (67.7)	0/0	0/0	10/20 (50.0)	26/41 (63.4)	27/32 (84.4)

*Resistance proportions are reported as no. resistant isolates/no. isolates tested. Blank cells indicate that no organisms were tested during that period and, thus, the proportion of resistant organisms is unknown. 3GC, third-generation cephalosporin; AMP–GEN, resistance to both ampicillin and gentamicin; CRO, ceftriaxone; FQ, fluoroquinolone; MDR, multidrug resistant; MAC/LIN, resistance to macrolides and/or lincosamides; MET, methicillin; VAN, vancomycin.  †*K. pneumoniae* is intrinsically resistant to AMP, and thus AMP–GEN resistance in *K. pneumoniae* isolates is equivalent to GEN resistance.

Resistance differed greatly among the 3 groups of *Salmonella* spp. The proportion of resistant isolates was highest for *Salmonella* Typhi: 95.7% (308/322) were fluoroquinolone resistant, 86.0% (270/314) multidrug resistant, and 85.0% (266/313) fluoroquinolone and multidrug resistant. The least resistant group was *Salmonella* Paratyphi A: 22.7% (10/44) of isolates were fluoroquinolone resistant and none were multidrug resistant (0/43). Resistance in nontyphoidal *Salmonella* spp. fell between that of *Salmonella* Typhi and Paratyphi A: 63.4% (26/41) of isolates were fluoroquinolone resistant, 23.1% (9/39) multidrug resistant, and 12.8% (5/39) fluoroquinolone and multidrug resistant. Only 1.6% (4/254) of *Salmonella* spp. isolates were ceftriaxone resistant.

In gram-positive GLASS organisms, approximately one third of *S. pneumoniae* isolates were macrolide/lincosamide resistant (29.7%, 49/165), half were penicillin resistant (50.7%, 73/144), and two thirds were multidrug resistant (67.8%, 63/93). Only 13.0% (24/185) of *S. aureus* isolates were methicillin resistant.

The most frequently isolated non-GLASS pathogen was *Burkholderia pseudomallei*, the causative agent of melioidosis (26.1%, 66), which was universally sensitive to the first-line drugs ceftazidime and co-trimoxazole (Table 2). Next was *Haemophilus influenzae*, for which approximately half of isolates were ampicillin resistant (53.6%, 30/56) and one third multidrug resistant (37.1%, 13/35), followed by *Enterobacter cloacae*, which had a similar resistance profile to *K. pneumoniae* and *E. coli*. The remaining non-GLASS pathogens (group A *Streptococcus*, *Pseudomonas aeruginosa*, and *Neisseria meningitides*) exhibited low-level resistance to the key antimicrobials reported.

**Table 2:** Resistance proportions by year of isolation for the 253 non-Global Antimicrobial Resistance Surveillance System pathogens isolated from children at Angkor Hospital for Children, Siem Reap, Cambodia, 2007–2016*

Pathogen, resistance type	No. isolates resistant/no. tested (%)	Year of isolation				
		2007–2008	2009–2010	2011–2012	2013–2014	2015–2016
*Burkholderia pseudomallei*	66	6	10	13	22	15
CAZ	0/66	0/6	0/10	0/13	0/22	0/15
TMP/SXT	0/61	0/2	0/10	0/12	0/22	0/15
*Haemophilus influenzae*	57	15	15	9	12	6
AMP	30/56 (53.6)	5/14 (35.7)	10/15 (66.7)	7/9 (77.8)	8/12 (66.7)	0/6
CRO	3/57 (5.3)	1/15 (6.7)	1/15 (6.7)	0/9	1/12 (8.3)	0/6
Multidrug	13/35 (37.1)	0/0	5/10 (50.0)	5/7 (71.4)	3/12 (25.0)	0/6
*Enterobacter cloacae*	42	2	6	8	17	9
AMP–GEN	19/42 (45.2)	1/2 (50.0)	5/6 (83.3)	5/8 (62.5)	6/17 (35.3)	2/9 (22.2)
3GC	34/42 (81.0)	1/2 (50.0)	5/6 (83.3)	7/8 (87.5)	14/17 (82.4)	7/9 (77.8)
Carbapenem	3/41 (7.3)	0/1	0/6	0/8	2/17 (11.8)	1/9 (11.1)
Multidrug	18/37 (48.6)	1/1 (100)	2/2 (100)	5/8 (62.5)	7/17 (41.2)	3/9 (33.3)
Group A *Streptococcus*	38	2	6	6	13	11
MAC/LIN	6/37 (16.2)	0/2	1/5 (20.0)	0/6	2/13 (15.4)	3/11 (27.3)
*Pseudomonas aeruginosa*	37	7	6	7	9	8
CAZ	4/34 (11.8)	0/4	1/6 (16.7)	1/7 (14.3)	2/9 (22.2)	0/8
Carbapenem	2/30 (6.7)	0/1	0/5	1/7 (14.3)	0/9	1/8 (12.5)
Multidrug	0/29	0/0	0/5	0/7	0/9	0/8
*Neisseria meningitidis*	13	6	3	0	2	2
CRO	1/13 (7.7)	0/6	1/3 (33.3)		0/2	0/2

*Resistance proportions have been reported as number of resistant isolates out of number of isolates tested. Blank cell indicates that no organisms were tested during that period and, thus, the proportion of resistant organisms is unknown. 3GC, third-generation cephalosporin; AMP–GEN, resistance to both ampicillin and gentamicin; CAZ, ceftazidime; CRO, ceftriaxone; MAC/LIN, resistance to macrolides and/or lincosamides; TMP/SXT, trimethoprim/sulfamethoxazole.

### AMR Time Trends 

**Figure 1:**
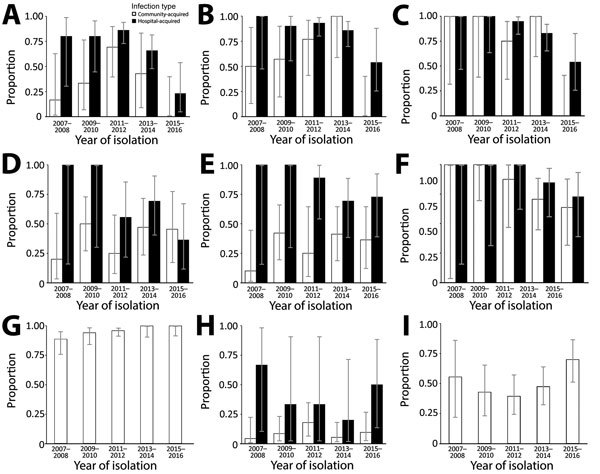
Antimicrobial resistance time trends, shown as proportion of resistant isolates from community-acquired and hospital-acquired infections, by year of isolation, in children at Angkor Hospital for Children, Siem Reap, Cambodia, 2007–2016. A) *Klebsiella pneumoniae* ampicillin–gentamicin resistance; B) *K. pneumoniae* third-generation cephalosporin resistance; C) *K. pneumoniae* multidrug resistance; D) *Escherichia coli* ampicillin–gentamicin resistance; E) *E. coli* third-generation cephalosporin resistance; F) *E. coli* multidrug resistance; G) *Salmonella enterica* serotype Typhi fluoroquinolone resistance; H) *Staphylococcus aureus* methicillin resistance; I) *Streptococcus pneumoniae* penicillin resistance. Isolates were defined as hospital-acquired if taken >48 hours after patient admission. Error bars indicate 95% CIs.

The most frequently isolated organisms were *K. pneumoniae, E. coli, Salmonella* Typhi, *S. aureus*, and *S. pneumoniae;* AMR time trends were heterogeneous (Tables 1, 3–5; Figure 1). *S. pneumoniae* penicillin resistance fluctuated over time; 55.6% (5/9) of isolates were resistant in 2007–2008, dropping to 41.0% (16/39) in 2011–2012 before rising to 68.8% (22/32) in 2015–2016. During 2011–2016, when we tested *S. pneumoniae* for multidrug resistance, the proportion of multidrug-resistant *S. pneumoniae* isolates increased from 50.0% to 84.4%. *Salmonella* Typhi fluoroquinolone resistance also increased over the study period, from 88.6% to 100%. Multivariable logistic regression analysis in which time was a continuous variable showed an increased probability of *Salmonella* Typhi fluoroquinolone resistance over time (adjusted odds ratio [aOR] 2.14, 95% CI 1.29–3.74; p = 0.005), although not statistically significant in the model when time was a factor (Technical Appendix Table 3, Figure 3). Conversely, during 2015–2016, the proportion of resistant *K. pneumoniae* isolates fell dramatically for most antimicrobials tested, a phenomenon not seen for *E. coli* (Technical Appendix Table 4). For *E. coli*, ampicillin–gentamicin and third-generation cephalosporin resistance remained stable at ≈50% with no evidence of significant change over time, as did rates of methicillin-resistant *S. aureus*, which remained low throughout the study period. The proportion of *K. pneumoniae* isolates from neonates peaked in 2011–2014 at 46%–50% before dropping in 2015–2016 to 35% (Technical Appendix Table 5), paralleling the change in the proportion of resistant isolates seen. To determine any subtle shifts in susceptibility, we examined changes in zone diameter distribution over time for *E. coli* and *K. pneumoniae* and found no clear trends (Technical Appendix Table 6, Figures 4, 5).

**Table 3:** Logistic regression analysis of resistance trends for the gram-negative Global Antimicrobial Resistance Surveillance System pathogens *Klebsiella pneumoniae* and *Escherichia coli* isolated from children at Angkor Hospital for Children, Siem Reap, Cambodia, 2007–2016*

Pathogen, resistance type, predictor variable	Univariable analysis			Multivariable analysis	
	OR (95% CI)	p value		OR (95% CI)	p value
*Klebsiella pneumoniae*					
AMP–GEN					
Year of isolation					
2007–2008	Ref	Ref		Ref	Ref
2009–2010	2.00 (0.42–10.03)	0.384		1.31 (0.23–7.88)	0.765
2011–2012	5.52 (1.41–22.90)	0.015		2.61 (0.58–12.45)	0.213
2013–2014	1.95 (0.51–7.80)	0.329		0.59 (0.12–2.85)	0.504
2015–2016	0.21 (0.03–1.12)	0.075		0.06 (0.01–0.41)	0.006
Patient age					
Nonneonate	Ref	Ref		Ref	Ref
Neonate†	5.63 (2.61–13.10)	<0.001		7.30 (2.75–22.47)	<0.001
Infection type‡					
Community-acquired	Ref	Ref		Ref	Ref
Hospital-acquired	3.87 (1.81–8.51)	<0.001		3.62 (1.42–9.58)	0.008
3GC					
Years of isolation					
2007–2008	Ref	Ref		Ref	Ref
2009–2010	1.22 (0.20–7.02)	0.823		0.87 (0.13–5.60)	0.881
2011–2012	3.13 (0.57–14.69	0.156		1.37 (0.23–7.16)	0.716
2013–2014	2.78 (0.49–13.93)	0.218		0.97 (0.15–5.58)	0.973
2015–2016	0.20 (0.03–0.94)	0.052		0.06 (0.01–0.39)	0.005
Patient age					
Nonneonate	Ref	Ref		Ref	Ref
Neonate	6.41 (2.32–22.70)	0.001		7.50 (2.16–35.00)	0.004
Infection type					
Community-acquired	Ref	Ref		Ref	Ref
Hospital-acquired	4.04 (1.76–9.44)	0.001		3.51 (1.27–10.12)	0.017
*Escherichia coli*					
AMP–GEN					
Years of isolation					
2007–2008	Ref	Ref		Ref	Ref
2009–2010	2.67 (0.63–12.75)	0.194		2.86 (0.66–14.13)	0.174
2011–2012	1.23 (0.28–5.86)	0.785		0.95 (0.20–4.75)	0.947
2013–2014	2.62 (0.67–11.63)	0.179		2.25 (0.53–10.72)	0.282
2015–2016	1.38 (0.33–6.50)	0.665		1.03 (0.22–5.11)	0.975
Patient age					
Nonneonate	Ref	Ref		Ref	Ref
Neonate	1.04 (0.42–2.57)	0.924		0.75 (0.27–2.01)	0.568
Infection type					
Community-acquired	Ref	Ref		Ref	Ref
Hospital-acquired	2.33 (1.04–5.33)	0.041		2.92 (1.21–7.44)	0.020
3GC					
Years of isolation					
2007–2008	Ref	Ref		Ref	Ref
2009–2010	3.00 (0.68–16.38)	0.165		4.04 (0.79–26.54)	0.112
2011–2012	3.30 (0.74–18.21)	0.134		2.47 (0.45–16.60)	0.319
2013–2014	3.43 (0.83–17.83)	0.105		3.07 (0.60–19.99)	0.201
2015–2016	3.60 (0.82–19.73)	0.106		2.44 (0.45–16.13)	0.319
Patient age					
Nonneonate	Ref	Ref		Ref	Ref
Neonate	0.92 (0.37–2.27)	0.861		0.41 (0.12–1.26)	0.131
Infection type					
Community-acquired	Ref	Ref		Ref	Ref
Hospital-acquired	7.50 (3.09–20.01)	<0.001		10.14 (3.70–32.14)	<0.001

*3GC, third-generation cephalosporin; AMP–GEN, resistance to both ampicillin and gentamicin; OR, odds ratio; ref, referent. †Neonate, 0–28 d of age. ‡Isolates were defined as hospital-acquired if taken >48 hours after admission.

**Table 5:** Logistic regression analysis of resistance trends for the gram-positive Global Antimicrobial Resistance Surveillance System pathogens *Staphylococcus aureus* and *Streptococcus pneumoniae* isolated from children at Angkor Hospital for Children, Siem Reap, Cambodia, 2007–2016*

Pathogen, resistance type, predictor variable	Univariable analysis			Multivariable analysis	
	OR (95% CI)	p value		OR ratio (95% CI)	p value
*S. aureus*					
Methicillin					
Year of isolation					
2007–2008	Ref	Ref		Ref	Ref
2009–2010	0.90 (0.18–4.93)	0.899		1.26 (0.23–7.59)	0.787
2011–2012	1.80 (0.47–8.90)	0.418		2.64 (0.62–14.48)	0.215
2013–2014	0.59 (0.10–3.42)	0.538		0.66 (0.10–4.19)	0.649
2015–2016	1.48 (0.35–7.61)	0.603		1.84 (0.39–10.47)	0.455
Patient age†					
Nonneonate	Ref	Ref		Ref	Ref
Neonate	0.18 (0.01–0.88)	0.094		0.14 (0.01–0.75)	0.064
Infection type‡					
Community-acquired	Ref	Ref		Ref	Ref
Hospital-acquired	6.21 (2.16–17.43)	<0.001		7.80 (2.51–24.81)	<0.001
*S. pneumoniae*§					
Penicillin					
Years of isolation					
2007–2008	Ref	Ref		Ref	Ref
2009–2010	0.60 (0.12–2.90)	0.525		0.70 (0.13–3.66)	0.669
2011–2012	0.52 (0.11–2.28)	0.385		0.42 (0.08–1.95)	0.269
2013–2014	0.72 (0.16–3.12)	0.663		0.77 (0.16–3.57)	0.737
2015–2016	1.87 (0.38–8.77)	0.424		1.89 (0.36–9.59)	0.436
Patient age, y					
>5	Ref	Ref		Ref	Ref
<5	3.40 (1.63- 7.39)	0.001		3.87 (1.77–8.83)	<0.001

*OR, odds ratio; ref, referent. †Ages are grouped into neonate (0–28 d) vs. nonneonate (>29 d) or <5 y vs. >5 y, as appropriate for the organism.  ‡Isolates were defined as hospital-acquired if taken >48 hours after patient admission. §Analysis included community-acquired *Streptococcus pneumoniae* isolates only (n = 160).

### AMR by Patient Age Group

**Figure 2:**
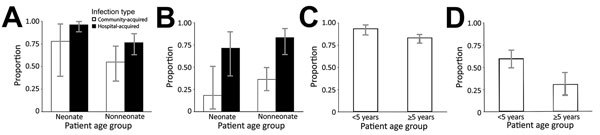
Antimicrobial resistance age trends, shown as proportion of resistant isolates from community-acquired and hospital-acquired infections, by patient age group, in children at Angkor Hospital for Children, Siem Reap, Cambodia, 2007–2016. A) *Klebsiella pneumoniae* third-generation cephalosporin resistance; B) *Escherichia coli* third-generation cephalosporin resistance; C) *Salmonella enterica* serotype Typhi multidrug resistance; D) *Streptococcus pneumoniae* penicillin resistance. Ages have been grouped into neonate (0–28 d) versus nonneonate (>29 d) or <5 years versus >5 y, as appropriate for the organism. Isolates were defined as hospital-acquired if taken >48 hours after admission. Error bars indicate 95% CIs.

Isolates from younger children were more often resistant to clinically important antimicrobials (Tables 3–5; Figure 2; Technical Appendix Tables 7–9). Multivariable logistic regression controlling for year of isolation and site of acquisition indicated that *K. pneumoniae* isolates from neonates were >7 times more likely to be resistant to the first-line treatment agents ampicillin–gentamicin (aOR 7.30, 95% CI 2.75–22.47) and third-generation cephalosporins (aOR 7.50, 95% CI 2.16–35.00) than were isolates from nonneonates. Similarly, *S. pneumoniae* isolates were more likely to be penicillin resistant (aOR 3.87, 95% CI 1.77–8.83) and *Salmonella* Typhi isolates more likely to be multidrug resistant (aOR 3.16, 95% CI 1.28- 9.57) among children <5 years of age than among those >5 years of age. 

### AMR by Site of Infection Acquisition

Approximately four fifths of included isolates were from community-acquired infections (1,089, 81.2%) and one fifth from hospital-acquired infections (252, 18.8%). In almost all instances, the proportion of hospital-acquired isolates resistant to a given antimicrobial was higher than that of community-acquired isolates (Technical Appendix Tables 10–12). *K. pneumoniae*, the main cause of hospital-acquired infections, was >3 times more likely to be resistant to ampicillin–gentamicin (aOR 3.62, 95% CI 1.42–9.58) and third-generation cephalosporins (aOR 3.51, 95% CI 1.27–10.12) in hospital-acquired isolates (Tables 3, 4). Increased likelihood of resistance among hospital-acquired isolates was also found for *E. coli* ampicillin–gentamicin and third-generation cephalosporin resistance and *S. aureus* methicillin resistance.

**Table 4:** Logistic regression analysis of resistance trends for the gram-negative Global Antimicrobial Resistance Surveillance System pathogen *Salmonella enterica* serovar Typhi isolated from children at Angkor Hospital for Children, Siem Reap, Cambodia, 2007–2016*

Resistance type, predictor variable	Univariable analysis			Multivariable analysis	
	OR (95% CI)	p value		OR (95% CI)	p value
Fluoroquinolone					
Year of isolation					
2007–2008	Ref	Ref		Ref	Ref
2009–2010	2.05 (0.47–10.51)	0.345		1.85 (0.42–9.59)	0.422
2011–2012	2.97 (0.82–10.37)	0.085		3.05 (0.83–10.74)	0.080
2013–2014	4.03 × 10^7^ (6.26 × 10^−45^–∞)	0.992		3.47 × 10^7^ (9.49 × 10^−44^–∞)	0.992
2015–2016	4.03 × 10^7^ (1.11 × 10^−43^–∞)	0.992		4.14 × 10^7^ (6.26 × 10^−43^–∞)	0.991
Patient age, y					
>5	Ref	Ref		Ref	Ref
<5	4.48 (0.87–82.12)	0.151		4.57 (0.87–84.30)	0.150
Multidrug					
Year of isolation					
2007–2008	Ref	Ref		Ref	Ref
2009–2010	1.57 (0.56–4.58)	0.395		1.45 (0.50–4.29)	0.491
2011–2012	4.32 (1.64–11.44)	0.003		4.55 (1.71–12.17)	0.002
2013–2014	2.26 (0.72–7.92)	0.175		2.08 (0.65–7.41)	0.228
2015–2016	0.91 (0.33–2.46)	0.850		0.95 (0.34–2.62)	0.927
Patient age, y					
>5	Ref	Ref		Ref	Ref
<5	2.94 (1.22–8.79)	0.029		3.16 (1.28- 9.57)	0.022

*OR, odds ratio; ref, referent.

### Outcomes

We analyzed patient outcomes for 129 admission episodes for community-acquired monomicrobial gram-negative bacteremia (Technical Appendix Figure 6). Of these, 63 (48.8%) isolates were resistant to third-generation cephalosporins and 34 admissions (26.4%) resulted in patient death. Isolates consisted of *E. coli* (48, 37.2%), *K. pneumoniae* (31, 24.0%), *A. baumannii* (29, 22.5%), and other pathogenic *Enterobacteriaceae* (21, 16.3%). Neonates accounted for 26.4% (34) of the cases; median age was 8.6 months (interquartile range [IQR] 0.8–29.2 months).

Children from whom third-generation cephalosporin-resistant bacteria were isolated were less likely than other patients to have received appropriate antimicrobial therapy (57% vs. 94%; p<0.001). If appropriate therapy was received, it was initiated later for children infected with third-generation–resistant than third-generation–sensitive organisms (2 days vs. 0 days after admission for those who survived [p<0.001]; 0.5 days vs. 0 days for those who died [p = 0.004]). Patients who died were younger (median age 1.4 vs. 10.2 months; p = 0.002), were more likely to have been admitted to an ICU (88% vs. 27%; p<0.001), stayed for a shorter time in hospital (3 vs. 8 days; p<0.001), and were more likely to have been infected with *Enterobacteriaceae* than *A. baumannii* (97% vs. 71%; p = 0.003) (Technical Appendix Table 13). *A. baumannii* infections were associated with high levels of third-generation cephalosporin resistance (90%) but a low mortality rate (3%) despite only 48% of patients having received appropriate antimicrobials. Conversely, *Enterobacteriaceae* infections were associated with a high mortality rate (33%) despite 84% of patients having received appropriate antimicrobials (Technical Appendix Table 14).

Multivariable logistic regression (Table 6) showed that third-generation cephalosporin resistance was associated with death (aOR 2.65, 95% CI 1.05–6.96; p = 0.042) and ICU admission (aOR 3.17, 95% CI 1.31–8.10; p = 0.013). Multivariable linear regression (Technical Appendix Table 15) controlling for the same variables also showed an association between length of hospital stay among survivors and third-generation cephalosporin resistance (1.69-fold increase, 95% CI 1.21–2.37). Third-generation cephalosporin resistance was associated with a 2.26-fold increase in hospital costs among survivors (95% CI 1.51–3.36) (Technical Appendix 16, 17). According to this model, the median cost per admission would have been US $432.00 (IQR $333.30–$613.90) if all infections were third-generation cephalosporin sensitive and US $974.10 (IQR $751.60–$1,384.30) if all infections were third-generation cephalosporin resistant.

**Table 6:** Multivariable logistic regression analysis of 129 hospital admission episodes for community-acquired monomicrobial gram-negative bacteremia from children at Angkor Hospital for Children, Siem Reap, Cambodia, 2007–2016*

Predictor variable	Death			ICU admission	
	OR (95% CI)	p value		OR (95% CI)	p value
Third-generation cephalosporin resistance	2.65 (1.05–6.96)	0.042		3.17 (1.31–8.10)	0.013
Neonate†	3.03 (1.14–8.31)	0.028		4.56 (1.83–12.16)	0.002
Male	0.81 (0.32–2.07)	0.659		0.81 (0.35–1.85)	0.616
*Enterobacteriaceae*‡	26.25 (4.43–511.1)	0.003		3.07 (1.05–9.67)	0.046
Malnourished§	2.11 (0.85–5.35)	0.111		2.19 (0.98–5.01)	0.059
Age <10 y	2.76 (0.40–56.29)	0.377		2.80 (0.60–20.70)	0.235

*Analysis used outcome (death or recovery) and ICU admission as the dependent variables. ICU, intensive care unit; OR, odds ratio. †0–28 d of age. ‡*Acinetobacter baumannii* n = 29; *Enterobacteriaceae* n = 100 (consisting of *Escherichia coli*, n = 48; *Klebsiella pneumoniae,* n = 31; other pathogenic *Enterobacteriaceae* [consisting of *Citrobacter*, *Enterobacter*, *Escherichia*, *Klebsiella*, *Morganella*, *Pantoea*, *Proteus*, and *Serratia* spp. n = 21]). §Children <10 y of age only.

## Discussion

In this hospitalized population of children in Cambodia, AMR levels were high, particularly among the gram-negative GLASS pathogens *K. pneumoniae*, *E. coli*, and *A. baumannii.* These organisms exhibited concerning resistance to WHO-recommended first-line sepsis treatment, emphasizing the urgent need for revised treatment guidelines (*4*). Few studies inform prevalence estimates of antimicrobial resistance in low- and middle-income countries in Asia, but compared with what is known, the high levels of gram-negative resistance reported here are not uncommon (*6*,*16*). For the gram-positive GLASS pathogens, *S. pneumoniae* resistance was broadly similar to that of the wider region (67.7% vs. 59.3% multidrug resistance, respectively) (*17*). Rates of methicillin-resistant *S. aureus* were comparatively lower; only 40.0% of hospital-acquired isolates were methicillin resistant compared with a regional average of 67.4% (*18*).

A major strength of this study is the observation of resistance trends over an extended period, something rarely possible in low- and middle-income countries because of lack of longstanding microbiology services. We found heterogeneous trends in resistance over time; resistance increased in some organisms (*Salmonella* Typhi) and decreased in others (*K. pneumoniae*). The most surprising temporal trend observed was a drop in the proportion of resistant *K. pneumoniae* isolates for most antimicrobials tested, in contrast to largely stable resistance levels in *E. coli*. For *K. pneumoniae* resistance by site of acquisition, in community-acquired isolates, resistance sharply declined in 2015–2016, perhaps suggesting loss of a plasmid coding for multiple resistance determinants. Confirming this trend will require a larger dataset from multiple sites in Cambodia and further analysis of the underlying resistance mechanisms at work using a method such as whole-genome sequencing. The genetic determinants of resistance in colonizing *K. pneumoniae* and *E. coli* isolates from this population have been reported elsewhere (CE Moore, CM Parry, P Turner, NPJ Day, N Stoesser, unpub data; N Stoesser, C Turner, P Turner, BS Cooper, unpub data), whereas whole-genome sequencing of invasive isolates is ongoing. Loss of antimicrobial selective pressure leading to declining resistance may result from changes in national/regional antimicrobial supply or lack of active drug in antimicrobials used (*19*).

The number of hospital-acquired *K. pneumoniae* isolates peaked during 2011–2012. This peak may be the result of a genuine rise in the rate of hospital-acquired infections or the increased rate of blood culture sampling compared with previous years. From 2011–2012 onward, the proportion of resistant hospital-acquired *K. pneumoniae* isolates declined. This drop may be linked to maturation of a hospitalwide infection-control program implemented in 2010 (*20*) and enforced by prospective hospital-acquired infection surveillance from 2015 onward (*21*) or to the clinical microbiology service operating since 2012 with a strong focus on antimicrobial drug stewardship. Indeed, a recent study of prescribing practices at this hospital found 84%–89% of antimicrobial drug prescriptions were appropriate (*22*). The apparent success of these interventions suggests that they could be useful for combating AMR in similar settings. The perceived temporal drop in *K. pneumoniae* resistance could also be attributable to changing proportions of isolates from neonates over time; 46%–50% of isolates were from neonates in 2011–2014, dropping to 35% in 2015–2016.

This study is unusual in that it directly compares different age groups of children, revealing AMR trends associated with age. Of note, the dominant pathogen in neonates, *K. pneumoniae*, was also more often resistant in neonates. For hospital-acquired isolates, this resistance may result from horizontal acquisition of resistant gram-negative organisms from hospital surfaces, as suggested by a recent multicenter study of sepsis in neonates (*2*). Indeed, colonization of neonates by resistant gram-negative organisms has been shown to be common at Angkor Hospital for Children and associated with subsequent invasive infection (*23*). For community-acquired isolates, vertical maternal transfer of resistant organisms may have a substantial role and is currently under investigation at this center.

Similarly, *Salmonella* Typhi from children <5 years of age was more often multidrug resistant than that from those >5 years of age. Isolates from younger children have greater genetic diversity (*24*), although how this diversity relates to increased AMR requires further investigation. In Cambodia, the most common indication for antimicrobial drug use is infections in children <5 years of age; thus, children in this age group may be exposed to more antimicrobial drugs, leading to greater resistance in organisms causing infection. That *S. pneumoniae* isolates were more often penicillin resistant in children <5 years of age is consistent with findings of previous work showing greater colonization of this age group by multidrug-resistant pneumococci (*25*). Vaccination may have a collateral benefit of reducing AMR (*26*), which suggests that it could be useful for combating *Salmonella* Typhi and *S. pneumoniae* resistance in low- and middle-income countries. Because 85% of *Salmonella* Typhi isolates are simultaneously fluoroquinolone resistant and multidrug resistant, few agents remain for treating typhoid in this population, placing even greater value on preventive measures such as vaccination. In January 2015, a 13-valent pneumococcal conjugate vaccine was introduced in Cambodia (*27*) with no catch-up campaign, meaning that only *S. pneumoniae* isolates from children born in or after December 2014 could have been affected, equating to 5 isolates in this dataset. Pneumococcal vaccination is thus unlikely to have had an appreciable effect on the AMR trends reported here.

The WHO Global Report on Surveillance identified a major gap in research comparing resource use in resistant versus nonresistant pathogens (*28*), an area that we addressed by demonstrating that resistance is associated with worse healthcare outcomes, including increased deaths and ICU admissions, delayed effective treatment, and more than doubled admission costs. Use of patient records allows these estimates to more closely reflect reality than modeled or ecologic analyses, although it is unclear whether this increased risk for adverse outcomes represents greater virulence, delayed treatment, or confounding. The observed outcome differences between *Enterobacteriaceae* and *A. baumannii* infections suggest either a true difference in virulence or that a proportion of *A. baumannii* isolates were contaminants, an uncertainty that highlights the difficulty of establishing the clinical significance of skin-colonizing organisms.

This study has several limitations. The data derive from a single nongovernmental hospital for children with limited numbers of isolates for some bacterial species; thus, trends and outcomes may not be representative of the wider region. The study was retrospective, and classification of community-acquired and hospital-acquired infections was limited by hospital database and clinical case note accuracy, meaning that some community-acquired infections may have actually been hospital-acquired infections. Widespread prehospitalization use of antimicrobials may have selected for resistant organisms (*29*). There were no restrictions to blood culture submission over time, but from early 2016 onward, clinicians were asked to focus on children requiring admission, which may have affected certain organisms (e.g., *Salmonella* Typhi). Microbiology practice variations over time meant that antimicrobial susceptibility testing was not consistent; however, we believe that the value of examining the evidence over a long period outweighed the effect that these variations may have had on results. For example, in 2009, the Clinical and Laboratory Standards Institute sensitivity zone size cutoffs for carbapenems and cephalosporins in *Enterobacteriaceae* increased, which could have resulted in a small number of isolates previously classed as sensitive being reclassified as resistant. The reported pre-2009 resistance levels are thus conservative and would not negate the downward resistance trends observed. In the outcome analysis, we did not consider prehospitalization factors, clinical diagnosis, and non-HIV/malnutrition co-occurring conditions because quantifying those could have introduced substantial reporting bias. Furthermore, our cost estimates may be higher than actual costs because we did not account for partial/shared doses and price fluctuations, suggesting that these cost estimates are most useful as a relative indication of cost burden.

In conclusion, the high rate of AMR in this setting of hospitalized children in Cambodia was associated with increased deaths and healthcare costs and threatens the effectiveness of first-line sepsis treatment. AMR represents a major threat to children’s health globally (*5*,*6*), yet there is a dearth of data for children in low-resource settings (*3*–*6*). By reporting a decade of continuous AMR surveillance data, this study fills a gap in the understanding of antimicrobial drug resistance in children in Cambodia. In the context of the current global drive to combat AMR and the goal of the Fleming Fund to improve surveillance in low- and middle-income countries, our study demonstrates the feasibility and utility of undertaking accurate long-term antimicrobial drug resistance surveillance in these countries. The methods used here are reproducible in similar low-resource settings.

Technical Appendix Supplementary methods and results for study of antimicrobial-resistant invasive bacteria in hospitalized children, Cambodia, 2007–2016.
