# Supplementary material for: Antimicrobial Resistance in Invasive Bacterial Infections in Hospitalized Children, Cambodia, 2007–2016
Source: Emerg Infect Dis. 2018 May;24(5):841–51. doi: 10.3201/eid2405.171830 (PMC5938766; doi:10.3201/eid2405.171830)
Supplement: Technical Appendix — Supplementary methods and results for study of antimicrobial-resistant invasive bacteria in hospitalized children, Cambodia, 2007–2016. [file 17-1830-Techapp-s1.pdf]

Article DOI: http://dx.doi.org/10.3201/eid2405.171830

Antimicrobial Resistance in Invasive Bacterial Infections in Hospitalized Children, Cambodia, 2007–2016

Technical Appendix

Methods

Classification of blood culture isolates

Coagulase-negative Staphylococci, *Micrococcus* species, and unspecified Gram-positive bacilli were classified as contaminants. Non-pathogenic environmental non-fermenting Gram-negatives were classified as of uncertain significance. All other bacterial species were considered as pathogens.

Management of Antimicrobial Susceptibility Testing data

Antimicrobial susceptibility testing (AST) results for BC isolates from 2007 to mid-2011 were interpreted as previously described (*1*). For BC isolates from mid-2011 onwards and CSFC isolates, disk-diffusion zone sizes and MIC results were available and re-interpreted using CLSI 2017 performance standards (*2*). Isolates classified as resistant, intermediate, or non-susceptible were analyzed as resistant. Changes in microbiology practice and guidelines meant species-specific AST panels varied over the study period. To account for this, pragmatic definitions of class resistance were used, and isolates only considered tested for multidrug resistant (MDR) status if they had undergone an appropriate minimum MDR test panel (Technical Appendix Table 1).

Technical Appendix Table 1. Definitions of antimicrobial resistance to be reported and minimum multidrug resistance (MDR) test panels for each organism/organism group*

Organism / Organism group	Resistance to be reported and definitions or resistance and sensitivity for antimicrobial classes	MDR test panel - minimum antimicrobial susceptibility tests required for isolate to be considered tested for MDR status
Salmonellae (*Salmonella* Typhi, *Salmonella* Paratyphi and Non-Typhoidal *Salmonellae*)	• Fluoroquinolone resistance = non-susceptibility to Ciprofloxacin (determined by Etest/MIC testing) and/or Nalidixic acid<br>• Ceftriaxone resistance<br>• Multidrug resistance = non-susceptibility to Ampicillin, Chloramphenicol and Co-trimoxazole	• Ampicillin<br>• Chloramphenicol<br>• Co-trimoxazole
Enterobacteriaceae (Klebsiella pneumoniae, Escherichia coli, Enterobacter cloacae)	• Ampicillin-Gentamicin resistance = non-susceptibility to both Ampicillin and Gentamicin<br>• 3GC (Ceftazidime or Ceftriaxone or Cefotaxime or ESBL testing or Cefpodoxime testing, plus one of the above 3GC tests if Cefpodoxime resistant)<br>• Carbapenem (Meropenem or Imipenem)<br>• Multidrug resistance = non-susceptibility to ≥1 agent in ≥3 antimicrobial classes tested, excluding intrinsic resistance (as documented in CLSI guideline M100-S27)	• Ampicillin<br>• Chloramphenicol<br>• Ciprofloxacin<br>• Co-trimoxazole<br>• Gentamicin<br>• 3GC<br>o Ceftazidime or Ceftriaxone or Cefotaxime or ESBL testing or Cefpodoxime testing<br>+ one of the above 3GC tests if Cefpodoxime resistant<br>• Carbapenem<br>o Meropenem or Imipenem
Acinetobacter baumannii	• 3GC Ceftazidime or Ceftriaxone or Cefotaxime or ESBL testing or Cefpodoxime testing, plus one of the above 3GC tests if Cefpodoxime resistant)<br>• Carbapenem (Meropenem or Imipenem)<br>• Multidrug resistance (as for *Enterobacteriaceae*)	• Ciprofloxacin<br>• Co-trimoxazole<br>• Gentamicin<br>• 3GC<br>o Ceftazidime or Ceftriaxone or Cefotaxime or Cefpodoxime testing<br>+ one of the above 3GC tests if Cefpodoxime resistant<br>• Carbapenem<br>o Meropenem or Imipenem
Staphylococcus aureus	• Methicillin resistance = non-susceptibility to Cefoxitin or Oxacillin<br>• Vancomycin resistance (as determined by Etest/MIC testing)<br>• Multidrug resistance excluding Methicillin resistance (as for *Enterobacteriaceae*, excluding Methicillin resistant organisms, which by definition are also multidrug resistant)	• Cefoxitin<br>• Ciprofloxacin<br>• Co-trimoxazole<br>• Erythromycin<br>• Gentamicin<br>• Penicillin
Streptococcus pneumoniae	• Oxacillin, plus Ceftriaxone Etest and Benzylpenicillin Etest if Oxacillin resistant<br>• Macrolide/Lincosamide (Clindamycin or Erythromycin)<br>• Multidrug resistance (as for *Enterobacteriaceae*)	• Chloramphenicol<br>• Co-trimoxazole<br>• Tetracycline<br>• Macrolide/Lincosamide<br>o Clindamycin or Erythromycin<br>• Oxacillin<br>+ Ceftriaxone Etest and Benzylpenicillin Etest if Oxacillin resistant
Neisseria meningitidis	• Ceftriaxone resistance	Not applicable
Burkholderia pseudomallei	• Ceftazidime resistance as defined previously by Wuthiekanun et al (*3*)<br>• Co-trimoxazole resistance as defined in CLSI guideline M45-A2 (*4*)	Not applicable
Haemophilus influenzae	• Ampicillin resistance<br>• Ceftriaxone resistance<br>• Multidrug resistance (as for *Enterobacteriaceae*)	• Ampicillin<br>• Ceftriaxone<br>• Chloramphenicol<br>• Ciprofloxacin<br>• Co-amoxiclav<br>• Co-trimoxazole
Pseudomonas aeruginosa	• Ceftazidime resistance<br>• Carbapenem (Meropenem or Imipenem)<br>• Multidrug resistance (as for *Enterobacteriaceae*)	• Ceftazidime<br>• Ciprofloxacin<br>• Gentamicin<br>• Carbapenem<br>o Meropenem or Imipenem
Group A *Streptococcus*	• Macrolide/Lincosamide resistance (as for *S. pneumoniae*)	Not applicable

*MIC, minimum inhibitory concentration; Amp-Gent, ampicillin and gentamicin; 3GC, 3rd generation cephalosporin; ESBL, extended spectrum beta lactamase; Co-trimoxazole, trimethoprim-sulfamethoxazole.

Technical Appendix Table 2. Proportion of blood cultures taken by patient age group and year for 2012-2016 (n = 27,021)*

	Specimens per year (% of year total)					
Category	2012	2013	2014	2015	2016	Total (% of total)
Patient age group						
0-28 days†	453 (9.1)	573 (10.9)	739 (12.5)	851 (13.4)	955 (21.2)	3571 (13.2)
1-59 months	2,780 (55.6)	3,030 (57.6)	3,596 (61.0)	3,653 (57.5)	2,547 (56.4)	15,606 (57.8)
≥5 years	1,769 (35.4)	1,653 (31.4)	1,558 (26.4)	1,854 (29.2)	1,010 (22.4)	7,844 (29.0)
Specimen year total	5,002	5,256	5,893	6,358	4,512	27,021

*Accurate patient age data for all blood cultures taken (including negative cultures) was only routinely recorded from 2012 onwards, and thus proportions have only been provided for years 2012-2016.
†Neonate was defined as age 0-28 days.

Technical Appendix Table 3. Logistic regression analysis of *Salmonella* Typhi fluoroquinolone resistance trends (n = 322)*

	Univariable analysis		Multivariable analysis		
Predictor variable	OR (95% CI)	p value	OR (95% CI)	p value	AIC
Model 1 (year of isolation as a factor)					
Year of isolation (factor)					
2007-2008	-	-	-	-	113
2009-2010	2.05 (0.47-10.51)	0.345	1.85 (0.42-9.59)	0.422	
2011-2012	2.97 (0.82-10.37)	0.085	3.05 (0.83-10.74)	0.080	
2013-2014	4.03×10^{7} (6.26×10^{-45}-NA)	0.992	3.47×10^{7} (9.49×10^{-44}-NA)	0.992	
2015-2016	4.03×10^{7} (1.11×10^{-43}-NA)	0.992	4.14×10^{7} (6.26×10^{-43}-NA)	0.991	
Patient age, y					
≥5	-	-	-	-	
<5	4.48 (0.87-82.12)	0.151	4.57 (0.87-84.30)	0.150	
Model 2 (year of isolation as a continuous variable)					
Year of isolation (continuous)					
2007-2016	2.12 (1.28- 3.72)	0.005	2.14 (1.29-3.74)	0.005	109
Patient age, y					
≥5	-	-	-	-	
<5	4.48 (0.87-82.12)	0.151	4.71 (0.90-86.83)	0.141	

*OR, odds ratio; AIC, Akaike information criterion.

Technical Appendix Table 4. Resistance proportions for all standard antimicrobials tested by year of isolation for *Klebsiella pneumoniae*, *Escherichia coli* and *Streptococcus pneumoniae**

	n isolates	Year of isolation				
Pathogen, resistance type	n resistant/ n tested (%)	2007-2008	2009-2010	2011-2012	2013-2014	2015-2016
Klebsiella pneumoniae	146	11	17	56	42	20
Amp-Gent†	90/145 (62.1)	5/11 (45.5)	10/16 (62.5)	46/56 (82.1)	26/42 (61.9)	3/20 (15.0)
3GC	115/146 (78.8)	8/11 (72.7)	13/17 (76.5)	50/56 (89.3)	37/42 (88.1)	7/20 (35.0)
Carbapenem	1/142 (0.7)	0/8 (0.0)	0/16 (0.0)	0/56 (0.0)	1/42 (2.4)	0/20 (0.0)
MDR	108/132 (81.8)	8/8 (100.0)	12/12 (100.0)	45/50 (90.0)	36/42 (85.7)	7/20 (35.0)
Co-amoxiclav	101/146 (69.2)	8/11 (72.7)	11/17 (64.7)	47/56 (83.9)	30/42 (71.4)	5/20 (25.0)
Chloramphenicol	65/136 (47.8)	7/8 (87.5)	11/13 (84.6)	26/53 (49.1)	19/42 (45.2)	2/20 (10.0)
Ciprofloxacin	86/142 (60.6)	7/11 (63.6)	8/16 (50.0)	39/53 (73.6)	27/42 (64.3)	5/20 (25.0)
Co-trimoxazole	111/143 (77.6)	8/11 (72.7)	12/15 (80.0)	49/55 (89.1)	35/42 (83.3)	7/20 (35.0)
Escherichia coli	107	12	22	21	30	22
Amp-Gent	50/106 (47.2)	4/12 (30.8)	12/21 (57.1)	8/21 (38.1)	17/30 (56.7)	9/22 (40.9)
Ampicillin	101/107 (94.4)	10/12 (83.3)	21/22 (95.5)	21/21 (100.0)	28/30 (93.3)	21/22 (95.5)
Gentamicin	51/106 (48.1)	4/12 (33.3)	12/21 (57.1)	8/21 (38.1)	18/30 (60.0)	9/22 (40.9)
3GC	53/107 (49.5)	3/12 (25.0)	11/22 (50.0)	11/21 (52.4)	16/30 (53.3)	12/22 (54.5)
Carbapenem	0/98 (0.0)	0/3 (0.0)	0/22 (0.0)	0/21 (0.0)	0/30 (0.0)	0/22 (0.0)
MDR	69/84 (82.1)	3/3 (100.0)	13/13 (100.0)	15/16 (93.8)	23/30 (76.7)	15/22 (68.2)
Co-amoxiclav	57/107 (53.3)	7/12 (58.3)	13/22 (59.1)	14/21 (66.7)	12/30 (40.0)	11/22 (50.0)
Chloramphenicol	38/86 (44.2)	3/3 (100.0)	8/13 (61.5)	10/18 (55.6)	11/30 (36.7)	6/22 (27.3)
Ciprofloxacin	48/105 (45.7)	6/12 (50.0)	10/22 (45.5)	8/19 (42.1)	15/30 (50.0)	9/22 (40.9)
Co-trimoxazole	89/105 (84.8)	10/12 (83.3)	17/20 (85.0)	20/21 (95.2)	24/30 (80.0)	18/22 (81.8)
Streptococcus pneumoniae	166	17	36	40	41	32
Penicillin	73/144 (50.7)	5/9 (55.6)	10/23 (43.5)	16/39 (41.0)	20/41 (48.8)	22/32 (68.8)
Mac/Linc	49/165 (29.7)	5/17 (29.4)	10/35 (28.6)	12/40 (30.0)	11/41 (26.8)	11/32 (34.4)
MDR	63/93 (67.7)	0/0	0/0	10/20 (50.0)	26/41 (63.4)	27/32 (84.4)
Chloramphenicol	44/130 (33.8)	1/4 (25.0)	8/17 (47.1)	8/36 (22.2)	17/41 (41.5)	10/32 (31.3)
Co-trimoxazole	101/134 (75.4)	1/1 (100.0)	15/20 (75.0)	23/40 (57.5)	33/41 (80.5)	29/32 (90.6)
Tetracycline	87/93 (93.5)	0/0	0/0	18/20 (90.0)	39/41 (95.1)	30/32 (93.8)
Ceftriaxone	6/134 (4.5)	0/9 (0.0)	1/23 (4.3)	1/37 (2.7)	0/41 (0.0)	4/24 (16.7)

*Resistance proportions have been reported as number of resistant isolates out of actual number of isolates tested. Amp-Gent, resistance of an isolate to both ampicillin and gentamicin; 3GC, 3rd generation cephalosporin; MDR, multidrug resistant; Co-amoxiclav, amoxicillin/clavulanic acid; Mac/Linc, resistance of an isolate to macrolides and/or lincosamides; Co-trimoxazole, trimethoprim-sulfamethoxazole.
†*Klebsiella pneumoniae* is intrinsically resistant to ampicillin, and thus ampicillin-gentamicin resistance in *K. pneumoniae* isolates is equivalent to gentamicin resistance.

Technical Appendix Table 5. Proportional numbers of *Klebsiella pneumoniae* isolates by patient age group and year of isolation (n = 146)

	Isolates per year of isolation (% of year group total)					
Category	2007-2008	2009-2010	2011-2012	2013-2014	2015-2016	Total (% of total)
Patient age group						
0-28 days*	1 (9.1)	5 (29.4)	26 (46.4)	21 (50.0)	7 (35.0)	60 (41.1)
1-59 months	7 (63.6)	10 (58.8)	24 (42.9)	17 (40.5)	10 (50.0)	68 (46.6)
≥5 years	3 (27.3)	2 (11.8)	6 (10.7)	4 (9.5)	3 (15.0)	18 (12.3)
Year group total	11	17	56	42	20	146

*Neonate was defined as age 0-28 days.

Technical Appendix Table 6. Mean disk diffusion zone of inhibition diameter size by year of isolation for *Klebsiella pneumoniae* and *Escherichia coli* isolates testing sensitive or resistant to Gentamicin, Ceftriaxone and Imipenem, 2012-2016*

Pathogen, resistance type	Year of isolation									
	2012		2013		2014		2015		2016	
	mean (SD)	n / tested	mean (SD)	n / tested	mean (SD)	n / tested	mean (SD)	n / tested	mean (SD)	n / tested
Klebsiella pneumoniae										
Gentamicin										
Sensitive	18.3 (1.0)	6/30	17.4 (1.8)	7/17	19.0 (3.7)	9/25	17.4 (1.3)	7/8	17.6 (1.1)	10/12
Resistant	7.7 (1.1)	24/30	8.1 (1.9)	10/17	8.1 (1.4)	16/25	10.0 (NA)	1/8	9.0 (1.4)	2/12
Ceftriaxone										
Sensitive	26.6 (1.3)	5/30	26.3 (1.2)	3/17	27.7 (2.5)	3/25	29.0 (2.8)	5/8	28.8 (1.8)	8/12
Resistant	6.8 (1.8)	25/30	8.0 (2.6)	14/17	9.4 (3.2)	22/25	7.0 (1.7)	3/8	9.0 (3.2)	4/12
Imipenem†										
Sensitive	27.8 (1.8)	30/30	27.5 (1.5)	17/17	26.9 (2.4)	24/25	27.9 (1.7)	8/8	27.1 (0.9)	7/7
Resistant		0/30		0/17	21.0 (NA)	1/25		0/8		0/7
Escherichia coli										
Gentamicin										
Sensitive	19.2 (4.3)	9/13	19.0 (1.4)	2/9	18.2 (1.5)	10/21	18.3 (1.6)	6/9	17.6 (1.1)	7/13
Resistant	7.3 (1.0)	4/13	7.0 (1.2)	7/9	7.7 (2.1)	11/21	6.7 (0.6)	3/9	6.7 (1.0)	6/13
Ceftriaxone										
Sensitive	26.2 (1.5)	5/13	29.2 (3.3)	5/9	27.7 (2.6)	9/21	29.8 (1.5)	6/9	29.3 (2.1)	4/13
Resistant	9.4 (4.3)	8/13	12.0 (6.5)	4/9	8.9 (4.0)	12/21	6.7 (1.2)	3/9	6.8 (1.2)	9/13
Imipenem†										
Sensitive	29.5 (2.4)	13/13	28.2 (2.0)	9/9	27.6 (2.4)	21/21	27.8 (1.6)	9/9	28.0 (1.1)	8/8
Resistant		0/13		0/9		0/21		0/9		0/8

*Zone of inhibition sizes were only available for complete years 2012-2016. SD, standard deviation.
†Note that at the end of 2016, carbapenem testing was switched from imipenem to meropenem. Thus 5 *Klebsiella pneumoniae* isolates and 5 *Escherichia coli* isolates from 2016 are not included in this table.

Technical Appendix Table 7. Resistance proportions by patient age group for Gram-negative Global Antimicrobial Resistance Surveillance System (GLASS) pathogens (n = 736)*

Pathogen, site of acquisition, resistance type	n isolates n resistant/n tested (%)	Patient age group		
		0-28 days†	1-59 months	≥5 years
Klebsiella pneumoniae				
CAI	40 (27.4)	9	21	10
Amp-Gent	15/39 (38.5)	6/9 (66.7)	6/20 (30.0)	3/10 (30.0)
3GC	24/40 (60.0)	7/9 (77.8)	12/21 (57.1)	5/10 (50.0)
Carbapenem	0/36 (0.0)	0/9 (0.0)	0/18 (0.0)	0/9 (0.0)
MDR	23/33 (69.7)	7/9 (77.8)	11/15 (73.3)	5/9 (55.6)
HAI	106 (72.6)	51	47	8
Amp-Gent	75/106 (70.8)	44/51 (86.3)	27/47 (57.4)	4/8 (50.0)
3GC	91/106 (85.8)	49/51 (96.1)	37/47 (78.7)	5/8 (62.5)
Carbapenem	1/106 (0.9)	0/51 (0.0)	1/47 (2.1)	0/8 (0.0)
MDR	85/99 (85.9)	45/48 (93.8)	35/44 (79.5)	5/7 (71.4)
Escherichia coli				
CAI	69 (64.5)	11	50	8
Amp-Gent	27/68 (39.7)	2/11 (18.2)	20/49 (40.8)	5/8 (62.5)
3GC	23/69 (33.3)	2/11 (18.2)	15/50 (30.0)	6/8 (75.0)
Carbapenem	0/60 (0.0)	0/10 (0.0)	0/42 (0.0)	0/8 (0.0)
MDR	37/47 (78.7)	4/8 (0.5)	26/32 (81.3)	7/7 (100.0)
HAI	38 (35.5)	14	19	5
Amp-Gent	23/38 (60.5)	10/14 (71.4)	10/19 (52.6)	3/5 (60.0)
3GC	30/38 (78.9)	10/14 (71.4)	15/19 (78.9)	5/5 (100.0)
Carbapenem	0/38 (0.0)	0/14 (0.0)	0/19 (0.0)	0/5 (0.0)
MDR	32/37 (86.5)	12/14 (85.7)	15/18 (83.3)	5/5 (100.0)
Acinetobacter baumannii				
CAI	44 (58.7)	11	20	13
3GC	39/44 (88.6)	9/11 (81.8)	18/20 (90.0)	12/13 (92.3)
Carbapenem	4/44 (9.1)	2/11 (18.2)	2/20 (10.0)	0/13 (0.0)
MDR	8/43 (18.6)	2/10 (20.0)	4/20 (20.0)	2/13 (15.4)
HAI	31 (41.3)	5	21	5
3GC	31/31 (100.0)	5/5 (100.0)	21/21 (100.0)	5/5 (100.0)
Carbapenem	6/30 (20.0)	0/5 (0.0)	4/20 (20.0)	2/5 (40.0)
MDR	13/28 (46.4)	2/5 (40.0)	9/18 (50.0)	2/5 (40.0)
Salmonella Typhi				
Total (all CAI)	323	0	80	243
FQ	308/322 (95.7)		79/80 (98.8)	229/242 (94.6)
MDR	270/314 (86.0)		74/79 (93.7)	196/235 (83.4)

Pathogen, site of acquisition, resistance type	n isolates n resistant/n tested (%)	Patient age group		
		0-28 days†	1-59 months	≥5 years
Salmonella Paratyphi A				
Total (all CAI)	44	0	3	41
FQ	10/44 (22.7)		1/3 (33.3)	9/41 (22.0)
MDR	0/43 (0.0)		0/3 (0.0)	0/40 (0.0)
Non-Typhoidal *Salmonellae*				
CAI	39 (95.1)	2	30	7
FQ	24/39 (61.5)	1/2 (50.0)	19/30 (63.3)	4/7 (57.1)
MDR	8/37 (21.6)	0/1 (0.0)	4/30 (13.3)	4/6 (66.7)
HAI	2 (4.9)	0	2	0
FQ	2/2 (100.0)		2/2 (100.0)	
MDR	1/2 (50.0)		1/2 (50.0)	

*Resistance proportions have been reported as number of resistant isolates out of actual number of isolates tested. CAI, community-acquired infection; HAI, hospital-acquired infection; Amp-Gent, resistance of an isolate to both ampicillin and gentamicin; 3GC, 3rd generation cephalosporin; MDR, multidrug resistant; FQ, fluoroquinolone.
†Neonate was defined as age 0-28 days.

Technical Appendix Table 8. Resistance proportions by patient age group for Gram-positive Global Antimicrobial Resistance Surveillance System (GLASS) pathogens (n = 352)*

Pathogen, site of acquisition, resistance type	n isolates n resistant/n tested (%)	Patient age group		
		0-28 days†	1-59 months	≥5 years
Staphylococcus aureus				
CAI	166 (89.2)	29	68	69
Methicillin	16/165 (9.7)	0/29 (0.0)	13/68 (19.1)	3/68 (4.4)
Vancomycin	0/5 (0.0)	0/0	0/2 (0.0)	0/3 (0.0)
MDR (excluding Methicillin resistance)	12/146 (8.2)	1/25 (4.0)	5/60 (8.3)	6/61 (9.8)
HAI	20 (10.8)	4	7	9
Methicillin	8/20 (40.0)	1/4 (25.0)	2/7 (28.6)	5/9 (55.6)
Vancomycin	0/4 (0.0)	0/1 (0.0)	0/2 (0.0)	0/1 (0.0)
MDR (excluding Methicillin resistance)	1/19 (5.3)	0/4 (0.0)	0/6 (0.0)	1/9 (11.1)
Streptococcus pneumoniae				
CAI	160 (96.4)	2	105	53
Penicillin	69/138 (50.0)	1/2 (50.0)	54/90 (60.0)	14/46 (30.4)
Mac/Linc	45/159 (28.3)	0/2 (0.0)	34/104 (32.7)	11/53 (20.8)
MDR	61/90 (67.8)	1/2 (50.0)	43/59 (72.9)	17/29 (58.6)
HAI	6 (3.6)	0	5	1
Penicillin	4/6 (66.7)		4/5 (80.0)	0/1 (0.0)
Mac/Linc	4/6 (66.7)		4/5 (80.0)	0/1 (0.0)
MDR	2/3 (66.7)		2/3 (66.7)	0/0

*Resistance proportions have been reported as number of resistant isolates out of actual number of isolates tested. CAI, community-acquired infection; HAI, hospital-acquired infection; MDR, multidrug resistant; Mac/Linc, resistance of an isolate to macrolides and/or lincosamides.
†Neonate was defined as age 0-28 days.

Technical Appendix Table 9. Resistance proportions by patient age group for non-Global Antimicrobial Resistance Surveillance System (non-GLASS) pathogens (n = 253)*

Pathogen, site of acquisition, resistance type	n isolates<br>n resistant/n tested (%)	Patient age group		
		0-28 days†	1-59 months	≥5 years
Burkholderia pseudomallei				
Total (all CAI)	66	3	36	27
Ceftazidime	0/66 (0.0)	0/3 (0.0)	0/36 (0.0)	0/27 (0.0)
Co-trimoxazole	0/61 (0.0)	0/3 (0.0)	0/32 (0.0)	0/26 (0.0)
Haemophilus influenzae				
CAI	56 (98.2)	2	49	5
Ampicillin	30/55 (54.5)	1/2 (50.0)	27/48 (56.3)	2/5 (40.0)
Ceftriaxone	3/56 (5.4)	0/2 (0.0)	2/49 (4.1)	1/5 (20.0)
MDR	13/34 (38.2)	0/2 (0.0)	12/29 (41.4)	1/3 (33.3)
HAI	1 (1.8)	0	1	0
Ampicillin	0/1 (0.0)		0/1 (0.0)	
Ceftriaxone	0/1 (0.0)		0/1 (0.0)	
MDR	0/1 (0.0)		0/1 (0.0)	
Enterobacter cloacae				
CAI	12 (28.6)	3	6	3
Amp-Gent	6/12 (50.0)	2/3 (66.7)	3/6 (50.0)	1/3 (33.3)
3GC	8/12 (66.7)	3/3 (100.0)	4/6 (66.7)	1/3 (33.3)
Carbapenem	0/12 (0.0)	0/3 (0.0)	0/6 (0.0)	0/3 (0.0)
MDR	5/10 (50.0)	2/3 (66.7)	2/5 (40.0)	1/2 (50.0)
HAI	30 (73.2)	15	12	3
Amp-Gent	13/30 (43.3)	8/15 (53.3)	4/12 (33.3)	1/3 (33.3)
3GC	26/30 (86.7)	14/15 (93.3)	10/12 (83.3)	2/3 (66.7)
Carbapenem	3/29 (10.3)	1/15 (6.7)	2/11 (18.2)	0/3 (0.0)
MDR	13/27 (48.1)	8/15 (53.3)	4/9 (44.4)	1/3 (33.3)
Group A Streptococcus				
CAI	37 (97.4)	17	16	4
Mac/Linc	5/36 (13.9)	0/16 (0.0)	5/16 (31.3)	0/4 (0.0)
HAI	1 (2.6)	0	0	1
Mac/Linc	1/1 (100.0)			1/1 (100.0)
Pseudomonas aeruginosa				
CAI	20 (54.1)	1	13	6
Ceftazidime	2/17 (11.8)	0/1 (0.0)	1/11 (9.1)	1/5 (20.0)
Carbapenem	0/14 (0.0)	0/0	0/10 (0.0)	0/4 (0.0)
MDR	0/13 (0.0)	0/0	0/10 (0.0)	0/3 (0.0)
HAI	17 (45.9)	5	11	1
Ceftazidime	2/17 (11.8)	0/5 (0.0)	2/11 (18.2)	0/1 (0.0)
Carbapenem	2/16 (12.5)	0/5 (0.0)	2/10 (20.0)	0/1 (0.0)
MDR	0/16 (0.0)	0/5 (0.0)	0/10 (0.0)	0/1 (0.0)
Neisseria meningitidis				
Total (all CAI)	13	1	10	2
Ceftriaxone	1/13 (7.7)	0/1 (0.0)	1/10 (10.0)	0/2 (0.0)

*Resistance proportions have been reported as number of resistant isolates out of actual number of isolates tested. CAI, community-acquired infection; HAI, hospital-acquired infection; Amp-Gent, resistance of an isolate to both ampicillin and gentamicin; 3GC, 3rd generation cephalosporin; MDR, multidrug resistant; Mac/Linc, resistance of an isolate to macrolides and/or lincosamides; Co-trimoxazole, trimethoprim-sulfamethoxazole.
†Neonate was defined as age 0-28 days.

Technical Appendix Table 10. Resistance proportions by year of isolation and site of acquisition for Gram-negative Global Antimicrobial Resistance Surveillance System (GLASS) pathogens (n = 736)*

Pathogen, site of acquisition, resistance type	n isolates<br>n resistant/n tested (%)	Year of isolation<br>2007-2008	2009-2010	2011-2012	2013-2014	2015-2016
Klebsiella pneumoniae						
CAI	40 (27.4)	6	7	13	7	7
Amp-Gent	15/39 (38.5)	1/6 (16.7)	2/6 (33.3)	9/13 (69.2)	3/7 (42.9)	0/7 (0.0)
3GC	24/40 (60.0)	3/6 (50.0)	4/7 (57.1)	10/13 (76.9)	7/7 (100.0)	0/7 (0.0)
Carbapenem	0/36 (0.0)	0/3 (0.0)	0/6 (0.0)	0/13 (0.0)	0/7 (0.0)	0/7 (0.0)
MDR	23/33 (69.7)	3/3 (100.0)	4/4 (100.0)	9/12 (75.0)	7/7 (100.0)	0/7 (0.0)
HAI	106 (72.6)	5	10	43	35	13
Amp-Gent	75/106 (70.8)	4/5 (80.0)	8/10 (80.0)	37/43 (86.0)	23/35 (65.7)	3/13 (23.1)
3GC	91/106 (85.8)	5/5 (100.0)	9/10 (90.0)	40/43 (93.0)	30/35 (85.7)	7/13 (53.8)
Carbapenem	1/106 (0.9)	0/5 (0.0)	0/10 (0.0)	0/43 (0.0)	1/35 (2.9)	0/13 (0.0)
MDR	85/99 (85.9)	5/5 (100.0)	8/8 (100.0)	36/38 (94.7)	29/35 (82.9)	7/13 (53.8)
Escherichia coli						
CAI	69 (64.5)	10	19	12	17	11
Amp-Gent	27/68 (39.7)	2/10 (20.0)	9/18 (50.0)	3/12 (25.0)	8/17 (47.1)	5/11 (45.5)
Ampicillin	64/69 (92.8)	8/10 (80.0)	18/19 (94.7)	12/12 (100.0)	16/17 (94.1)	10/11 (90.9)
Gentamicin	28/68 (41.2)	2/10 (20.0)	9/18 (50.0)	3/12 (25.0)	9/17 (52.9)	5/11 (45.5)
3GC	23/69 (33.3)	1/10 (10.0)	8/19 (42.1)	3/12 (25.0)	7/17 (41.2)	4/11 (36.4)
Carbapenem	0/60 (0.0)	0/1 (0.0)	0/19 (0.0)	0/12 (0.0)	0/17 (0.0)	0/11 (0.0)
MDR	37/47 (78.7)	1/1 (100.0)	10/10 (100.0)	7/8 (87.5)	12/17 (70.6)	7/11 (63.6)
HAI	38 (35.5)	2	3	9	13	11
Amp-Gent	23/38 (60.5)	2/2 (100.0)	3/3 (100.0)	5/9 (55.6)	9/13 (69.2)	4/11 (36.4)
Ampicillin	37/38 (97.4)	2/2 (100.0)	3/3 (100.0)	9/9 (100.0)	12/13 (92.3)	11/11 (100.0)
Gentamicin	23/38 (60.5)	2/2 (100.0)	3/3 (100.0)	5/9 (55.6)	9/13 (69.2)	4/11 (36.4)
3GC	30/38 (78.9)	2/2 (100.0)	3/3 (100.0)	8/9 (88.9)	9/13 (69.2)	8/11 (72.7)
Carbapenem	0/38 (0.0)	0/2 (0.0)	0/3 (0.0)	0/9 (0.0)	0/13 (0.0)	0/11 (0.0)
MDR	32/37 (86.5)	2/2 (100.0)	3/3 (100.0)	8/8 (100.0)	11/13 (84.6)	8/11 (72.7)
Acinetobacter baumannii						
CAI	44 (58.7)	2	4	17	16	5
3GC	39/44 (88.6)	2/2 (100.0)	3/4 (75.0)	14/17 (82.4)	16/16 (100.0)	4/5 (80.0)
Carbapenem	4/44 (9.1)	1/2 (50.0)	0/4 (0.0)	2/17 (11.8)	1/16 (6.3)	0/5 (0.0)
MDR	8/43 (18.6)	1/2 (50.0)	1/4 (25.0)	4/16 (25.0)	2/16 (12.5)	0/5 (0.0)
HAI	31 (41.3)	0	3	13	11	4
3GC	31/31 (100.0)		3/3 (100.0)	13/13 (100.0)	11/11 (100.0)	4/4 (100.0)
Carbapenem	6/30 (20.0)		1/2 (50.0)	3/13 (23.1)	2/11 (18.2)	0/4 (0.0)
MDR	13/28 (46.4)		1/2 (50.0)	5/11 (45.5)	6/11 (54.5)	1/4 (25.0)
Salmonella Typhi						
Total (all CAI)	323	44	51	146	40	42
FQ	308/322 (95.7)	39/44 (88.6)	48/51 (94.1)	139/145 (95.9)	40/40 (100.0)	42/42 (100.0)
Ceftriaxone	1/173 (0.6)	0/44 (0.0)	1/21 (4.8)	0/26 (0.0)	0/40 (0.0)	0/42 (0.0)
MDR	270/314 (86.0)	31/41 (75.6)	39/47 (83.0)	134/144 (93.1)	35/40 (87.5)	31/42 (73.8)
Salmonella Paratyphi A						
Total (all CAI)	44	3	0	0	35	6
FQ	10/44 (22.7)	3/3 (100.0)			4/35 (11.4)	3/6 (50.0)
Ceftriaxone	0/44 (0.0)	0/3 (0.0)			0/35 (0.0)	0/6 (0.0)
MDR	0/43 (0.0)	0/2 (0.0)			0/35 (0.0)	0/6 (0.0)
Non-Typhoidal *Salmonellae*						
CAI	39 (95.1)	7	4	7	9	12
FQ	24/39 (61.5)	4/7 (57.1)	2/4 (50.0)	4/7 (57.1)	6/9 (66.7)	8/12 (66.7)
Ceftriaxone	3/35 (8.6)	0/7 (0.0)	0/4 (0.0)	1/3 (33.3)	0/9 (0.0)	2/12 (16.7)
MDR	8/37 (21.6)	3/7 (42.9)	1/2 (50.0)	2/7 (28.6)	2/9 (22.2)	0/12 (0.0)
HAI	2 (4.9)	0	0	0	0	2
FQ	2/2 (100.0)					2/2 (100.0)
Ceftriaxone	0/2 (0.0)					0/2 (0.0)
MDR	1/2 (50.0)					1/2 (50.0)

*Resistance proportions have been reported as number of resistant isolates out of actual number of isolates tested. CAI, community-acquired infection; HAI, hospital-acquired infection; Amp-Gent, resistance of an isolate to both ampicillin and gentamicin; 3GC, 3rd generation cephalosporin; MDR, multidrug resistant; FQ, fluoroquinolone.

†*Klebsiella pneumoniae* is intrinsically resistant to ampicillin, and thus ampicillin-gentamicin resistance in *K. pneumoniae* isolates is equivalent to gentamicin resistance.

Technical Appendix Table 11. Resistance proportions by year of isolation and site of acquisition for Gram-positive Global Antimicrobial Resistance Surveillance System (GLASS) pathogens (n = 352)*

Pathogen, site of acquisition, resistance type	n isolates n resistant/n tested (%)	Year of isolation				
		2007-2008	2009-2010	2011-2012	2013-2014	2015-2016
Staphylococcus aureus						
CAI	166 (89.2)	23	35	40	37	31
Methicillin	16/165 (9.7)	1/23 (4.3)	3/35 (8.6)	7/39 (17.9)	2/37 (5.4)	3/31 (9.7)
Vancomycin	0/5 (0.0)	0/0	0/0	0/0	0/2 (0.0)	0/3 (0.0)
MDR (excluding Methicillin resistance)	12/146 (8.2)	7/22 (31.8)	3/27 (11.1)	1/29 (3.4)	0/37 (0.0)	1/31 (3.2)
HAI	20 (10.8)	3	3	3	5	6
Methicillin	8/20 (40.0)	2/3 (66.7)	1/3 (33.3)	1/3 (33.3)	1/5 (20.0)	3/6 (50.0)
Vancomycin	0/4 (0.0)	0/0	0/0	0/0	0/1 (0.0)	0/3 (0.0)
MDR (excluding Methicillin resistance)	1/19 (5.3)	1/3 (33.3)	0/3 (0.0)	0/2 (0.0)	0/5 (0.0)	0/6 (0.0)
Streptococcus pneumoniae						
CAI	160 (96.4)	17	34	39	40	30
Penicillin	69/138 (50.0)	5/9 (55.6)	9/21 (42.9)	15/38 (39.5)	19/40 (47.5)	21/30 (70.0)
Mac/Linc	45/159 (28.3)	5/17 (29.4)	9/33 (27.3)	11/39 (28.2)	10/40 (25.0)	10/30 (33.3)
MDR	61/90 (67.8)	0/0	0/0	10/20 (50.0)	25/40 (62.5)	26/30 (86.7)
HAI	6 (3.6)	0	2	1	1	2
Penicillin	4/6 (66.7)		1/2 (50.0)	1/1 (100.0)	1/1 (100.0)	1/2 (50.0)
Mac/Linc	4/6 (66.7)		1/2 (50.0)	1/1 (100.0)	1/1 (100.0)	1/2 (50.0)
MDR	2/3 (66.7)		0/0	0/0	1/1 (100.0)	1/2 (50.0)

*Resistance proportions have been reported as number of resistant isolates out of actual number of isolates tested. CAI, community-acquired infection; HAI, hospital-acquired infection; MDR, multidrug resistant; Mac/Linc, resistance of an isolate to macrolides and/or lincosamides.

Technical Appendix Table 12. Resistance proportions by year of isolation and by site of acquisition for non-Global Antimicrobial Resistance Surveillance System (non-GLASS) pathogens (n = 253)*

Pathogen, site of acquisition, resistance type	n isolates n resistant/n tested (%)	Year of isolation				
		2007-2008	2009-2010	2011-2012	2013-2014	2015-2016
Burkholderia pseudomallei						
Total (all CAI)	66	6	10	13	22	15
Ceftazidime	0/66 (0.0)	0/6 (0.0)	0/10 (0.0)	0/13 (0.0)	0/22 (0.0)	0/15 (0.0)
Co-trimoxazole	0/61 (0.0)	0/2 (0.0)	0/10 (0.0)	0/12 (0.0)	0/22 (0.0)	0/15 (0.0)
Haemophilus influenzae						
CAI	56 (98.2)	15	15	9	12	5
Ampicillin	30/55 (54.5)	5/14 (35.7)	10/15 (66.7)	7/9 (77.8)	8/12 (66.7)	0/5 (0.0)
Ceftriaxone	3/56 (5.4)	1/15 (6.7)	1/15 (6.7)	0/9 (0.0)	1/12 (8.3)	0/5 (0.0)
MDR	13/34 (38.2)	0/0	5/10 (50.0)	5/7 (71.4)	3/12 (25.0)	0/5 (0.0)
HAI	1 (1.8)	0	0	0	0	1
Ampicillin	0/1 (0.0)					0/1 (0.0)
Ceftriaxone	0/1 (0.0)					0/1 (0.0)
MDR	0/1 (0.0)					0/1 (0.0)
Enterobacter cloacae						
CAI	12 (28.6)	1	4	3	3	1
Amp-Gent	6/12 (50.0)	1/1 (100.0)	3/4 (75.0)	2/3 (66.7)	0/3 (0.0)	0/1 (0.0)
3GC	8/12 (66.7)	1/1 (100.0)	3/4 (75.0)	3/3 (100.0)	1/3 (33.3)	0/1 (0.0)
Carbapenem	0/12 (0.0)	0/1 (0.0)	0/4 (0.0)	0/3 (0.0)	0/3 (0.0)	0/1 (0.0)
MDR	5/10 (50.0)	1/1 (100.0)	2/2 (100.0)	2/3 (66.7)	0/3 (0.0)	0/1 (0.0)
HAI	30 (73.2)	1	2	5	14	8
Amp-Gent	13/30 (43.3)	0/1 (0.0)	2/2 (100.0)	3/5 (60.0)	6/14 (42.9)	2/8 (25.0)
3GC	26/30 (86.7)	0/1 (0.0)	2/2 (100.0)	4/5 (80.0)	13/14 (92.9)	7/8 (87.5)
Carbapenem	3/29 (10.3)	0/0	0/2 (0.0)	0/5 (0.0)	2/14 (14.3)	1/8 (12.5)
MDR	13/27 (48.1)	0/0	0/0	3/5 (60.0)	7/14 (50.0)	3/8 (37.5)
Group A Streptococcus						
CAI	37 (97.4)	2	6	6	13	10
Mac/Linc	5/36 (13.9)	0/2 (0.0)	1/5 (20.0)	0/6 (0.0)	2/13 (15.4)	2/10 (20.0)
HAI	1 (2.6)	0	0	0	0	1
Mac/Linc	1/1 (100.0)					1/1 (100.0)
Pseudomonas aeruginosa						
CAI	20 (54.1)	6	5	2	3	4
Ceftazidime	2/17 (11.8)	0/3 (0.0)	1/5 (20.0)	1/2 (50.0)	0/3 (0.0)	0/4 (0.0)
Carbapenem	0/14 (0.0)	0/1 (0.0)	0/4 (0.0)	0/2 (0.0)	0/3 (0.0)	0/4 (0.0)

Pathogen, site of acquisition, resistance type	n isolates n resistant/n tested (%)	Year of isolation 2007-2008	2009-2010	2011-2012	2013-2014	2015-2016
MDR	0/13 (0.0)	0/0	0/4 (0.0)	0/2 (0.0)	0/3 (0.0)	0/4 (0.0)
HAI	17 (45.9)	1	1	5	6	4
Ceftazidime	2/17 (11.8)	0/1 (0.0)	0/1 (0.0)	0/5 (0.0)	2/6 (33.3)	0/4 (0.0)
Carbapenem	2/16 (12.5)	0/0	0/1 (0.0)	1/5 (20.0)	0/6 (0.0)	1/4 (25.0)
MDR	0/16 (0.0)	0/0	0/1 (0.0)	0/5 (0.0)	0/6 (0.0)	0/4 (0.0)
Neisseria meningitidis						
Total (all CAI)	13	6	3	0	2	2
Ceftriaxone	1/13 (7.7)	0/6 (0.0)	1/3 (33.3)		0/2 (0.0)	0/2 (0.0)

*Resistance proportions have been reported as number of resistant isolates out of actual number of isolates tested. CAI, community-acquired infection; HAI, hospital-acquired infection; Amp-Gent, resistance of an isolate to both ampicillin and gentamicin; 3GC, 3rd generation cephalosporin; MDR, multidrug resistant; Mac/Linc, resistance of an isolate to macrolides and/or lincosamides; Co-trimoxazole, trimethoprim-sulfamethoxazole.

Technical Appendix Table 13. Univariable logistic regression analysis of demographic characteristics, clinical characteristics and outcomes, compared by resistance and by outcome, for admission episodes due to community-acquired monomicrobial Gram-negative bacteremia (n = 129)*

Category	Overall	3GC Resistance Sensitive (n = 66)	3GC Resistance Resistant (n = 63)	3GC Resistance p value	Outcome Survived (n = 95)	Outcome Died (n = 34)	Outcome p value
**Demographic characteristics**							
**Median age in months (IQR)**	8.6 (0.8-29.2)	4.5 (0.4-20.7)	11.9 (1.2-63.0)	0.037	10.2 (1.5-42.6)	1.4 (0.1-11.7)	0.002
**Neonate (%)†**	34 (26%)	19 (29%)	15 (24%)	0.659	19 (20%)	15 (44%)	0.012
**Male (%)**	72 (56%)	38 (58%)	34 (54%)	0.814	51 (54%)	21 (62%)	0.540
**Clinical characteristics**							
**Malnourished (%)‡**	53 (41%)	28 (42%)	25 (40%)	0.891	33 (35%)	20 (59%)	0.025
***Enterobacteriaceae* infection§ (%)**	100 (78%)	63 (96%)	37 (59%)	<0.001	67 (71%)	33 (97%)	0.003
**HIV positive (%)**	1 (1%)	0 (0%)	1 (2%)	-	0 (0%)	1 (3%)	-
**3GC resistance (%)**	63 (49%)	-	-	-	46 (48%)	17 (50%)	1
**Outcomes**							
**Death (%)**	34 (26%)	17 (26%)	17 (27%)	1	-	-	-
**ICU admission (%)**	56 (43%)	25 (38%)	31 (49%)	0.263	26 (27%)	30 (88%)	<0.001
**Appropriate treatment received (%)¶**	98 (76%)	62 (94%)	36 (57%)	<0.001	74 (78%)	24 (71%)	0.534
**Median days to treatment (IQR)#**	0 (0-1.0)	-	-	-	0 (0-2)	0 (0 – 0)	0.087
**Survived**	0 (0-2.0)	0 (0-0)	2 (0.3-3.8)	<0.001	-	-	-
**Died**	0 (0-0)	0 (0-0)	0.5 (0-1.8)	0.004	-	-	-
**Median length of stay (IQR)****	7.0 (4.0-10.0)	-	-	-	8.0 (6.0-12.5)	3.0 (2.0-4.8)	<0.001
**Survived**	8.0 (6.0-12.5)	8.0 (5.0-11.0)	9.0 (6.3-18.0)	0.179	-	-	-
**Died**	3.0 (2.0-4.8)	2.0 (1.0-4.0)	4.0 (2.0-7.0)	0.030	-	-	-

***Percentages are shown as a percentage of the column total. *P*-values were calculated using the chi-squared test with Yates correction for categorical variables, and the Mann-Whitney-Wilcoxon test for continuous variables. 3GC, 3rd generation cephalosporin; IQR, interquartile range; HIV, human immunodeficiency virus; ICU, intensive care unit.**
†Neonate was defined as age 0-28 days and non-neonate as age ≥29 days.
‡Malnutrition in children aged under ten years was defined as per WHO AnthroPlus software (*5*). Lack of height measurements meant it was not possible to classify malnutrition in children aged over ten years.
§*Acinetobacter baumannii* n = 29, *Enterobacteriaceae* n = 100 (consisting of *Escherichia coli* n = 48, *Klebsiella pneumoniae* n = 31, other pathogenic ***Enterobacteriaceae* consisting of *Citrobacter*, *Enterobacter*, *Escherichia*, *Klebsiella*, *Morganella*, *Pantoea*, *Proteus*, and *Serratia* species n = 21).**
¶Appropriate treatment was defined as receipt of an antimicrobial to which the organism was susceptible.
#Time to appropriate treatment was defined as days between day of admission and day of receipt.
**Length of stay was calculated separately for discharged patients and patients who died, taking day of admission as day one.

Technical Appendix Table 14. Comparison of outcomes between *Enterobacteriaceae* infections and *Acinetobacter baumannii* infections in admission episodes due to community-acquired monomicrobial Gram-negative bacteremia (n = 129)*

Outcome	***Enterobacteriaceae* (n=100)**	***Acinetobacter baumannii* (n=29)**
3GC resistance	37 (37%)	26 (90%)
Death (%)	33 (33%)	1 (3%)
ICU admission (%)	46 (46%)	10 (35%)
Appropriate treatment received (%)†	84 (84%)	14 (48%)
Median days to treatment (IQR)‡		
Survived	0 (0-1.0)	0 (0-4)
Died	0 (0-0)	0 (0-0)
Median length of stay (IQR)§		
Survived	9.0 (7.0-15.0)	7.0 (4.8-10.3)
Died	3.0 (2.0-5.0)	3.0 (3.0-3.0)

*Percentages are shown as a percentage of the column total. 3GC, 3rd generation cephalosporin; ICU, intensive care unit; IQR, interquartile range.
†Appropriate treatment was defined as receipt of an antimicrobial to which the organism was susceptible.
‡Time to appropriate treatment was defined as days between day of admission and day of receipt.
§Length of stay was calculated separately for discharged patients and patients who died, taking day of admission as day one.

Technical Appendix Table 15. Length of stay in survivors: linear regression analysis of admission episodes due to community-acquired monomicrobial Gram-negative bacteremia, taking length of stay in survivors as the dependent variable (n = 129)*

Predictor variable	**Log coefficients (95% CI)**	**Coefficients (95% CI)**	**p value**
3GC resistance	0.52 (0.19-0.86)	1.69 (1.21-2.37)	0.003
Neonate†	0.54 (0.17-0.91)	1.72 (1.19-2.49)	0.005
Male	-0.17 (-0.48-0.14)	0.84 (0.62-1.15)	0.280
Enterobacteriaceae infection‡	0.52 (0.14-0.89)	1.68 (1.15-2.44)	0.008
Malnourished§	0.34 (0.03-0.65)	1.40 (1.03-1.92)	0.032
Age under 10 years	-0.008 (-0.49-0.47)	0.99 (0.62-1.60)	0.972

*The first column shows the coefficients from the log-transformed model, while the second column shows the coefficients that have been back-transformed and are now on a multiplicative scale. 3GC, 3rd generation cephalosporin.
†Neonate was defined as age 0-28 days and non-neonate as age ≥29 days.
‡*Acinetobacter baumannii* n = 29, *Enterobacteriaceae* n = 100 (consisting of *Escherichia coli* n = 48, *Klebsiella pneumoniae* n = 31, other pathogenic *Enterobacteriaceae* consisting of *Citrobacter*, *Enterobacter*, *Escherichia*, *Klebsiella*, *Morganella*, *Pantoea*, *Proteus*, and *Serratia* species n = 21).
§Malnutrition in children aged under ten years was defined as per WHO AnthroPlus software (*5*). Lack of height measurements meant it was not possible to classify malnutrition in children aged over ten years.

Technical Appendix Table 16. Total admission cost in survivors: Linear regression analysis of admission episodes due to community-acquired monomicrobial Gram-negative bacteremia, taking total admission cost in survivors as the dependent variable (n = 129)*

Predictor variable	**Log coefficient (95% CI)**	**Coefficient (95% CI)**	**p value**
3GC resistance	0.81 (0.42-1.21)	2.26 (1.51-3.36)	<0.001
Neonate†	1.16 (0.72-1.60)	3.20 (2.06-4.96)	<0.001
Male	-0.26 (-0.63-0.11)	0.77 (0.54-1.11)	0.164
Enterobacteriaceae infection‡	0.81 (0.37-1.25)	2.25 (1.44-3.51)	0.001
Malnourished§	0.37 (0.007-0.74)	1.45 (1.01-2.10)	0.046
Age under 10 years	-0.06 (-0.63-0.51)	0.94 (0.54-1.66)	0.835

*Total cost of admission defined as the sum of the cost of stay and cost of antimicrobials. The first column shows coefficients from the log-transformed model, while the second column shows coefficients that have been back-transformed, which are now on a multiplicative scale. 3GC, 3rd generation cephalosporin.
†Neonate was defined as age 0-28 days and non-neonate as age ≥29 days.
‡*Acinetobacter baumannii* n = 29, *Enterobacteriaceae* n = 100 (consisting of *Escherichia coli* n = 48, *Klebsiella pneumoniae* n = 31, other pathogenic *Enterobacteriaceae* consisting of *Citrobacter*, *Enterobacter*, *Escherichia*, *Klebsiella*, *Morganella*, *Pantoea*, *Proteus*, and *Serratia* species n = 21).
§Malnutrition in children aged under ten years was defined as per WHO AnthroPlus software (*5*). Lack of height measurements meant it was not possible to classify malnutrition in children aged over ten years.

Technical Appendix Table 17. Median costs of admission episodes due to community-acquired monomicrobial Gram-negative bacteremia (n = 129)*

**Cost**	**Total**	**3GC sensitive**	**3GC resistant**
Antibiotic costs	11.31 (5.97-91.30)	8.80 (5.88-18.08)	14.85 (6.67-574.90)
Cost of stay	500.00 (350.00-1010.00)	450.00 (350.00-909.00)	500.00 (350.00-1246.00)
Total cost	515.4 (354.80-1247.00)	458.0 (355.30-915.00)	804.8 (354.60-1831.00)

**Acinetobacter baumannii* n = 29, *Enterobacteriaceae* n = 100 (consisting of *Escherichia coli* n = 48, *Klebsiella pneumoniae* n = 31, other pathogenic *Enterobacteriaceae* consisting of *Citrobacter*, *Enterobacter*, *Escherichia*, *Klebsiella*, *Morganella*, *Pantoea*, *Proteus*, and *Serratia* species n = 21). Costs are listed as median cost (interquartile range). The cost of stay excluded costs of all drugs (antimicrobials and non-antimicrobials). All costs are in 2017 US $. Costs are only reported for the cases that recovered so that death does not confer a cost advantage. 3GC, 3rd generation cephalosporin.

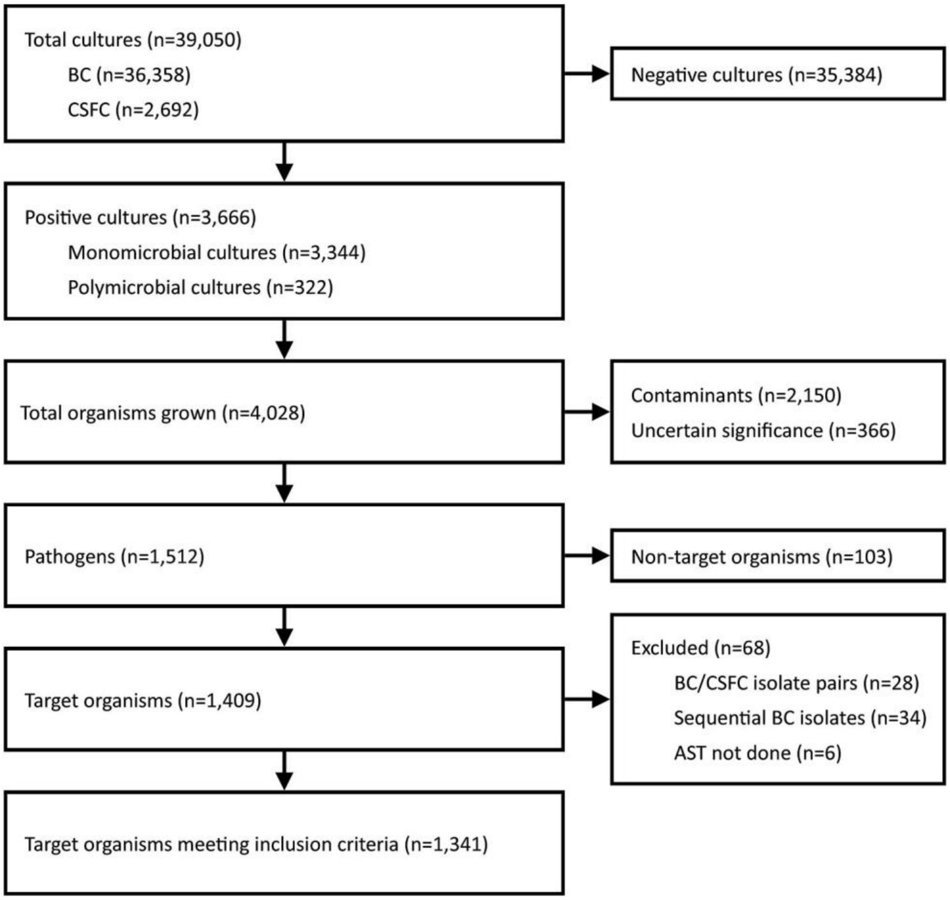

Technical Appendix Figure 1. Study profile. BC, blood culture; CSFC, cerebrospinal fluid culture; AST, antimicrobial susceptibility testing.

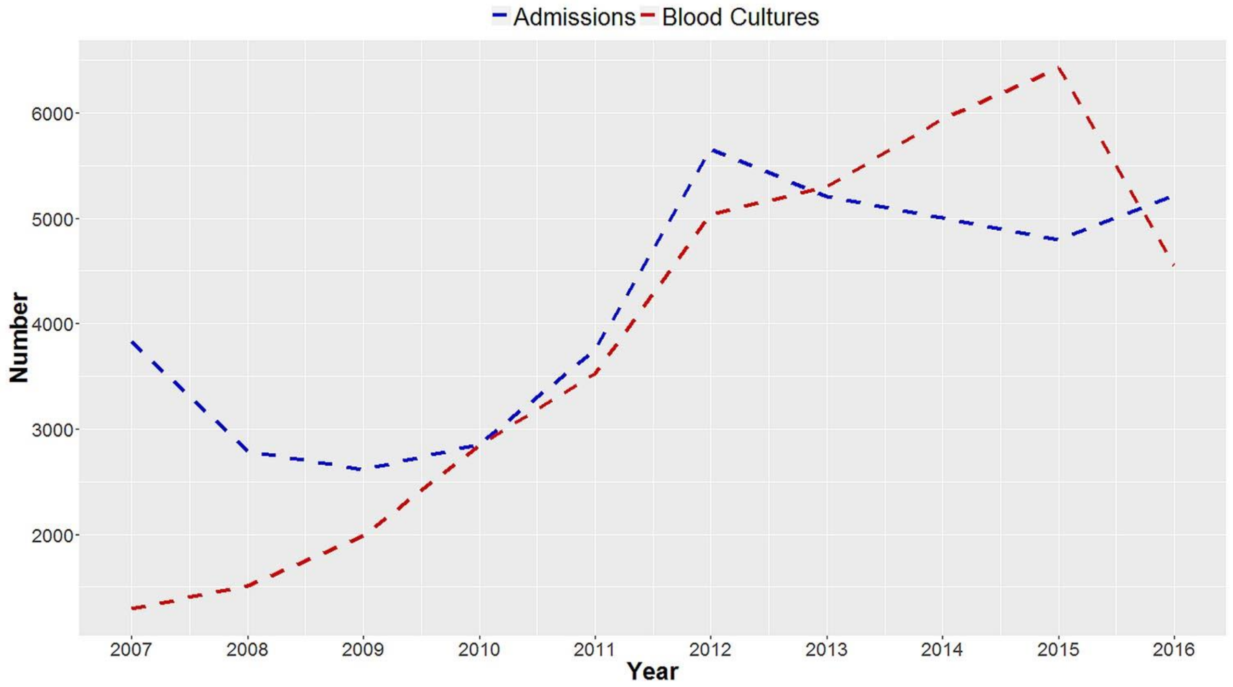

Technical Appendix Figure 2. Inpatient department admissions and blood cultures sent over time.

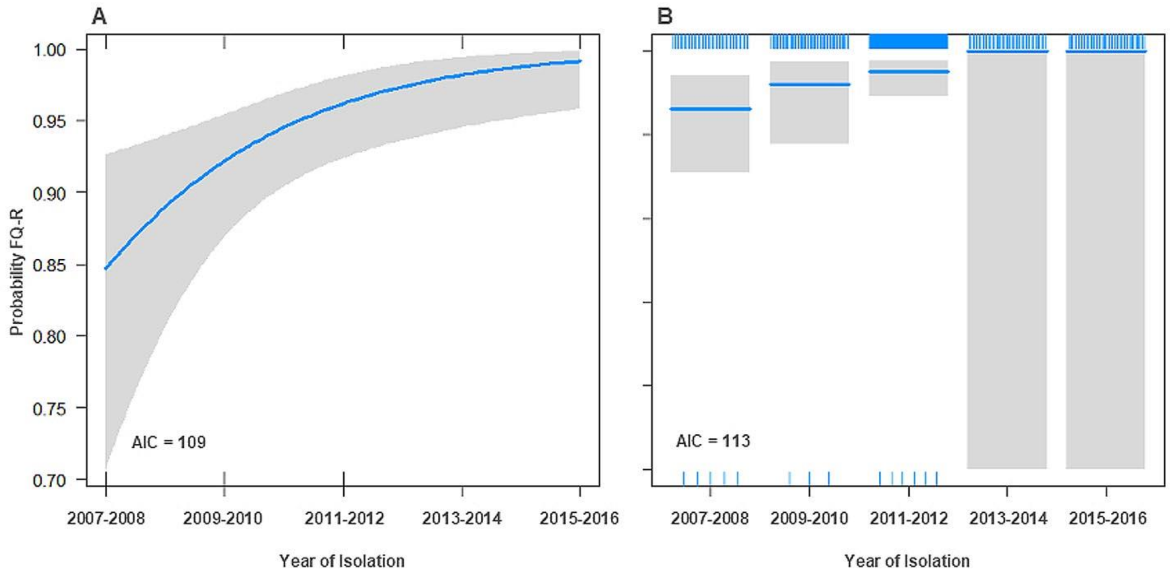

Technical Appendix Figure 3. Predicted probability of fluoroquinolone resistant *Salmonella* Typhi isolates by Year of Isolation, from multivariable logistic regression models with time modelled as a continuous variable (A) or a factor (B). FQ-R, fluoroquinolone resistance; AIC, Akaike Information Criterion.

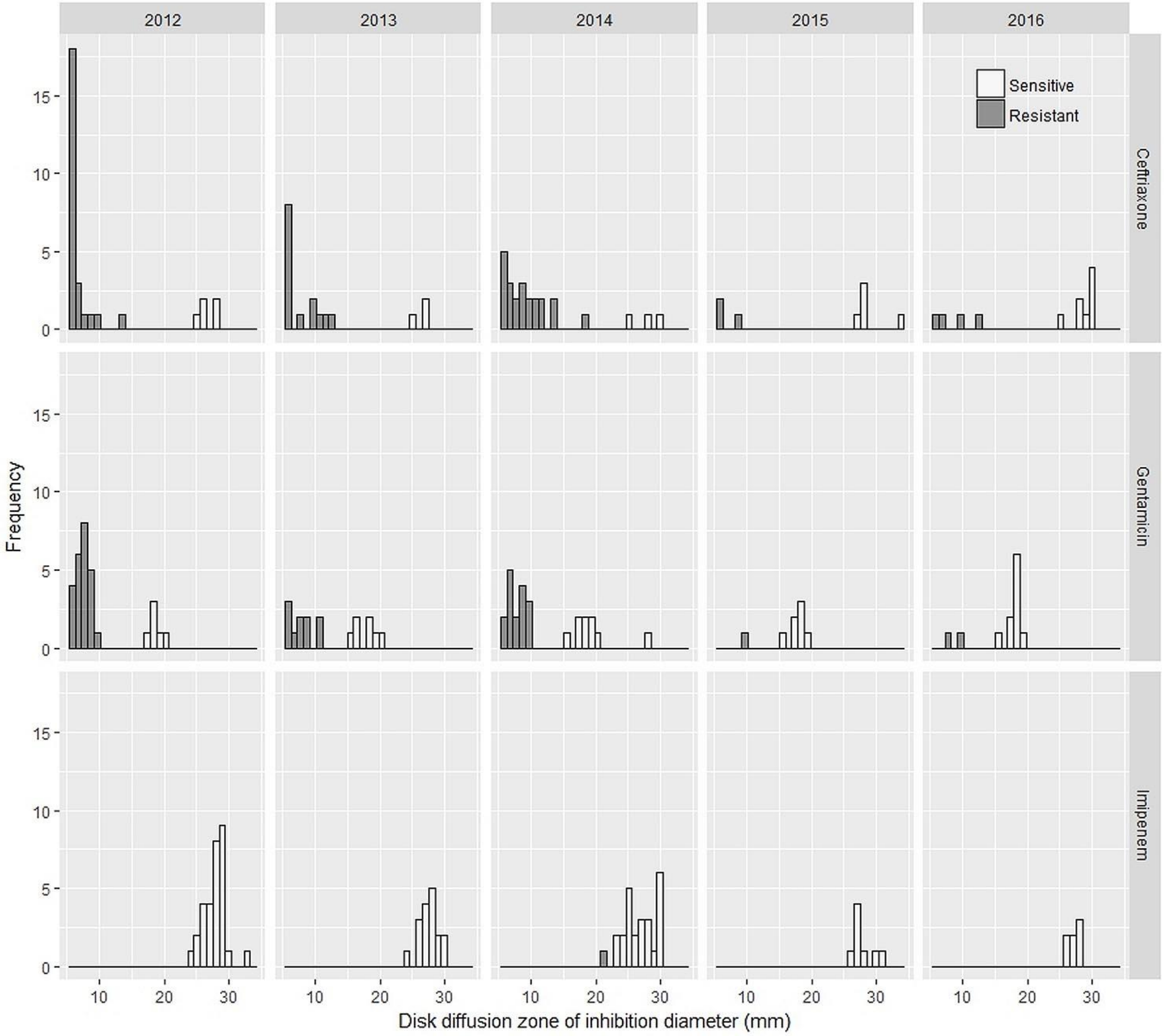

Technical Appendix Figure 4. Histogram of disk diffusion zone of inhibition diameter sizes by year for *Klebsiella pneumoniae* isolates testing sensitive or resistant to Gentamicin, Ceftriaxone and Imipenem, 2012-2016.

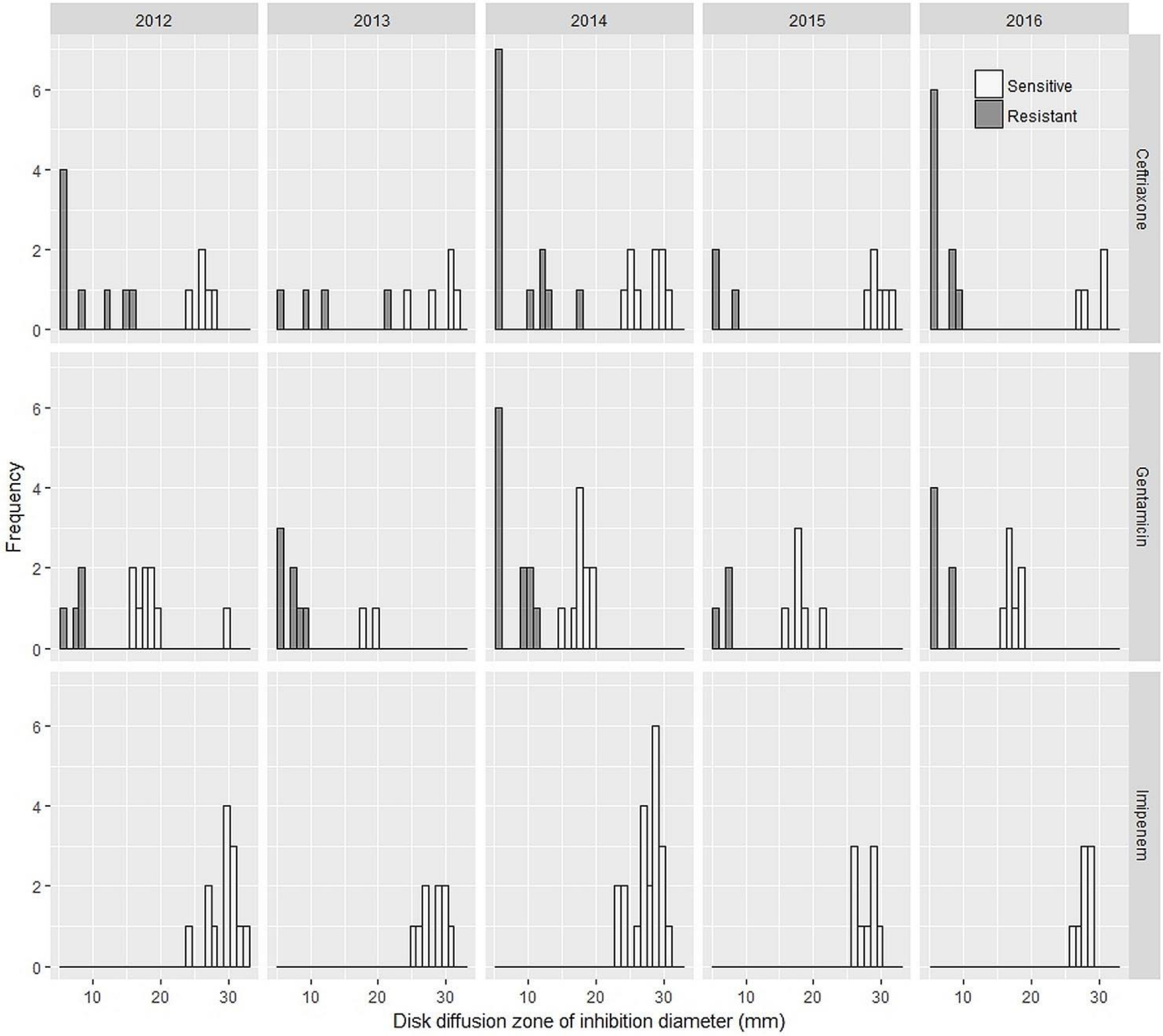

Technical Appendix Figure 5. Histogram of disk diffusion zone of inhibition diameter sizes by year for *Escherichia coli* isolates testing sensitive or resistant to Gentamicin, Ceftriaxone and Imipenem, 2012-2016.

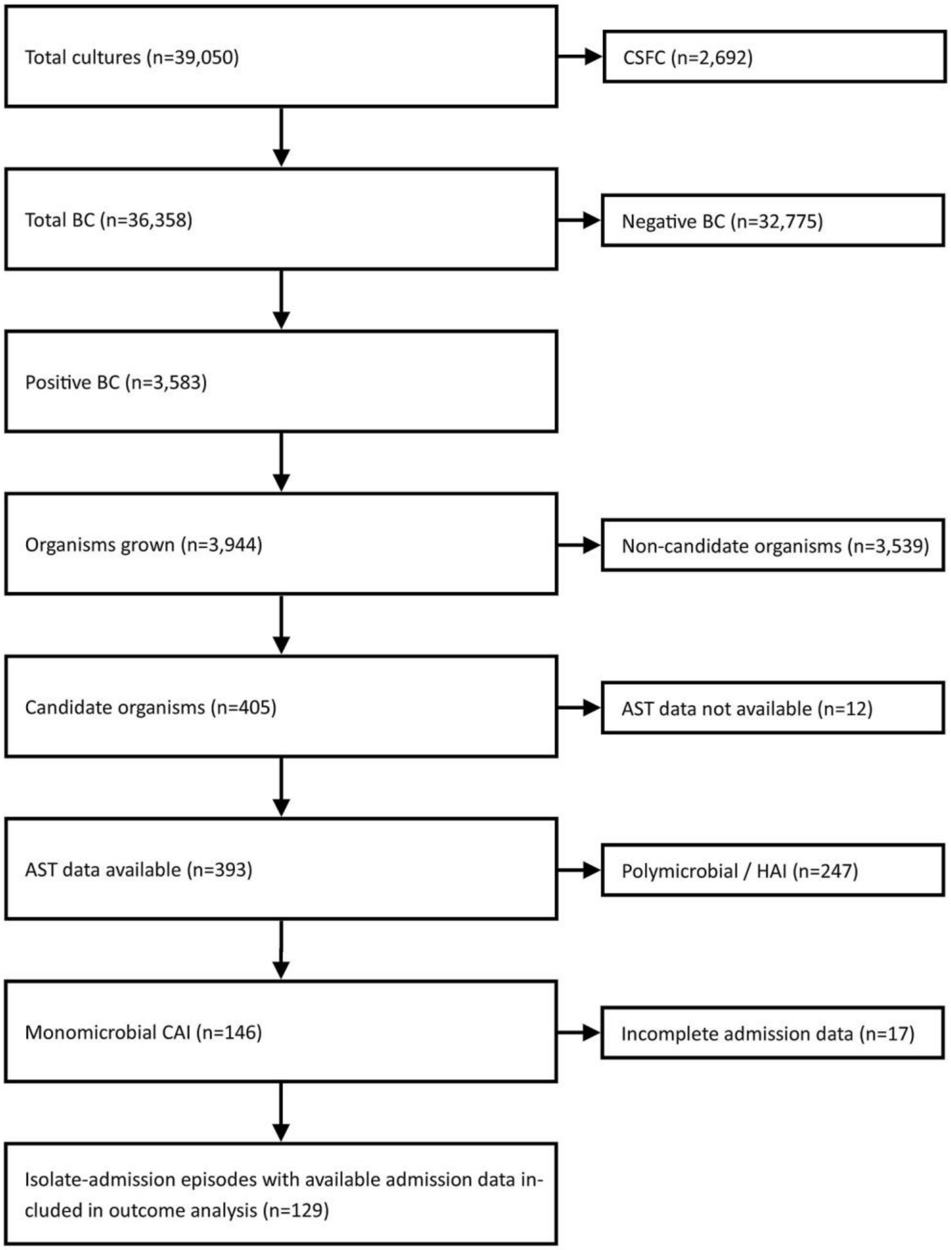

Technical Appendix Figure 6. Profile of isolate-admission episodes included in outcome analysis. BC, blood culture; CSFC, cerebrospinal fluid culture; AST, antimicrobial susceptibility testing; HAI, hospital-acquired infection; CAI, community-acquired infection.

References

1. Stoesser N, Moore CE, Pocock JM, An KP, Emary K, Carter M, et al. Pediatric bloodstream infections in Cambodia, 2007 to 2011. Pediatr Infect Dis J. 2013;32:e272–6. PubMed http://dx.doi.org/10.1097/INF.0b013e31828ba7c6

2. Clinical and Laboratory Standards Institute. Performance Standards for Antimicrobial Disk Susceptibility Tests. Twenty-Seventh Informational Supplement.:M100–S27. Wayne (PA): The Institute; 2017.

3. Wuthiekanun V, Amornchai P, Saiprom N, Chantratita N, Chierakul W, Koh GC, et al. Survey of antimicrobial resistance in clinical *Burkholderia pseudomallei* isolates over two decades in Northeast Thailand. Antimicrob Agents Chemother. 2011;55:5388–91. PubMed http://dx.doi.org/10.1128/AAC.05517-11

4. Clinical and Laboratory Standards Institute (CLSI). M45-A2 Methods for Antimicrobial Dilution and Disk Susceptibility Testing of Infrequently Isolated or Fastidious Bacteria; Approved Guideline - Second Edition. Wayne (PA): The Institute; 2010.

5. World Health Organization. AnthroPlus for personal computers: software for assessing growth of the world's children and adolescents. Geneva: The Organization; 2009.
